# Gene therapy of *Csf2ra* deficiency in mouse fetal monocyte precursors restores alveolar macrophage development and function

**DOI:** 10.1172/jci.insight.152271

**Published:** 2022-04-08

**Authors:** Fengqi Li, Katarzyna Maria Okreglicka, Federica Piattini, Lea Maria Pohlmeier, Christoph Schneider, Manfred Kopf

**Affiliations:** 1 Institute of Molecular Health Sciences, Department of Biology, ETH Zurich, Zurich, Switzerland.; 2 Institute of Physiology, University of Zurich, Zurich, Switzerland.

**Keywords:** Immunology, Gene therapy, Influenza, Macrophages

## Abstract

Tissue-resident macrophage-based immune therapies have been proposed for various diseases. However, generation of sufficient numbers that possess tissue-specific functions remains a major handicap. Here, we showed that fetal liver monocytes cultured with GM-CSF (CSF2-cFLiMo) rapidly differentiated into a long-lived, homogeneous alveolar macrophage–like population in vitro. CSF2-cFLiMo retained the capacity to develop into bona fide alveolar macrophages upon transfer into *Csf2ra^–/–^* neonates and prevented development of alveolar proteinosis and accumulation of apoptotic cells for at least 1 year in vivo. CSF2-cFLiMo more efficiently engrafted empty alveolar macrophage niches in the lung and protected mice from severe pathology induced by respiratory viral infection compared with transplantation of macrophages derived from BM cells cultured with M-CSF (CSF1-cBMM) in the presence or absence of GM-CSF. Harnessing the potential of this approach for gene therapy, we restored a disrupted *Csf2ra* gene in fetal liver monocytes and demonstrated their capacity to develop into alveolar macrophages in vivo. Altogether, we provide a platform for generation of immature alveolar macrophage–like precursors amenable for genetic manipulation, which will be useful to dissect alveolar macrophage development and function and for pulmonary transplantation therapy.

## Graphical abstract

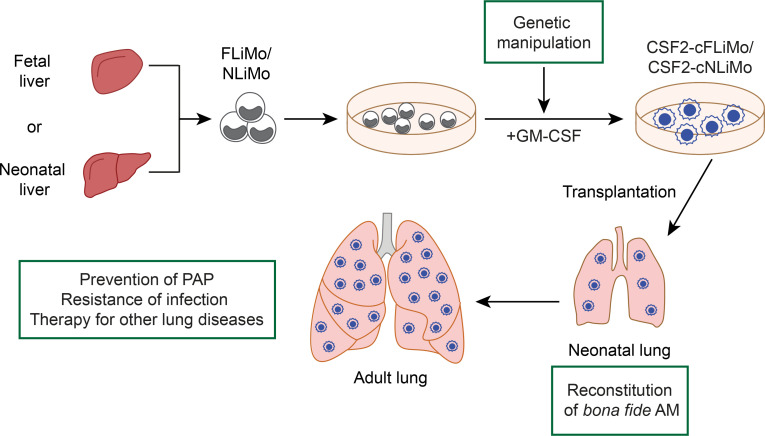

## Introduction

Tissue-resident macrophages comprise a variety of heterogeneous, tissue-adapted macrophage subsets that display a multitude of tissue-specific functions in homeostasis and disease (1, 2). Alveolar macrophages, the resident macrophage population in the lung, play important roles in host defense to pulmonary infections and noninflammatory clearance of inhaled particles (3). In the steady state, alveolar macrophages are required for catabolism of surfactant and removal of apoptotic cells that otherwise accumulate in the alveoli and result in impaired air exchange (4). Alveolar macrophages are derived from fetal liver monocytes, which seed the lung during late embryogenesis. Final maturation and expansion occur upon induction of the transcription factor PPARγ by GM-CSF perinatally (5, 6). Mature alveolar macrophages can be maintained life-long through self-renewal (7, 8). Accordingly, absence of PPARγ, GM-CSF, or 1 of the 2 GM-CSF receptor subunits (CSF2R α and β) in mice results in abrogated alveolar macrophage development and pulmonary alveolar proteinosis (PAP) (5, 6, 9–11). Similarly, mutations in the *CSF2RA* and *CSF2RB* genes in humans result in defective alveolar macrophage development and hereditary pulmonary alveolar proteinosis, which is associated with long-term respiratory insufficiency and high susceptibility to microbial infection (12–14).

It has been shown that pulmonary transplantation of BM-derived macrophages (BMMs) or induced pluripotent stem cell–derived macrophages to *Csf2rb^–/–^* mice can prevent development of PAP (15–18). Moreover, transplantation of human CD34^+^ cell–derived macrophage progenitors or induced pluripotent stem cell–derived macrophages have been shown to ameliorate hereditary PAP in a humanized mouse model (15, 19). However, whether these transplanted macrophages resemble bona fide self-renewing alveolar macrophages with functional capacities beyond surfactant clearance remains unclear. Here, we have established a biologically relevant model to study alveolar macrophage development and function by gene editing in vitro and assessment of functional consequences in vivo. By culturing purified fetal liver monocytes with GM-CSF (CSF2-cFLiMo), we were able to generate high numbers of alveolar macrophage–like cells that can be kept proliferating like stem cells for at least up to a year, in vitro, and possess the ability to terminally differentiate and restore alveolar macrophage development and function upon pulmonary transplantation to *Csf2ra^–/–^* neonates. CSF2-cFLiMo–derived alveolar macrophages resemble bona fide alveolar macrophages in gene expression profile, surface markers, and functional capacities, including potent self-renewal capacity, clearance of surfactant, and efferocytosis of apoptotic cells during influenza virus infection. Transplanted CSF2-cFLiMo more efficiently engrafted empty alveolar macrophage niches and protected mice from influenza infection compared with transplanted BM cells cultured with M-CSF (CSF1-cBMM) or CSF1/CSF2-cBMM. Finally, *Csf2ra* retroviral gene transfer into *Csf2ra*-deficient fetal liver monocytes restored their potential to develop into bona fide alveolar macrophages in vivo. Altogether, this study provides a potent platform to investigate genes involved in alveolar macrophage development and function, as well as a platform for therapeutic approaches.

## Results

### In vitro GM-CSF–differentiated fetal liver monocytes give rise to self-renewing cells with alveolar macrophage–like phenotype.

**Figure 1:**
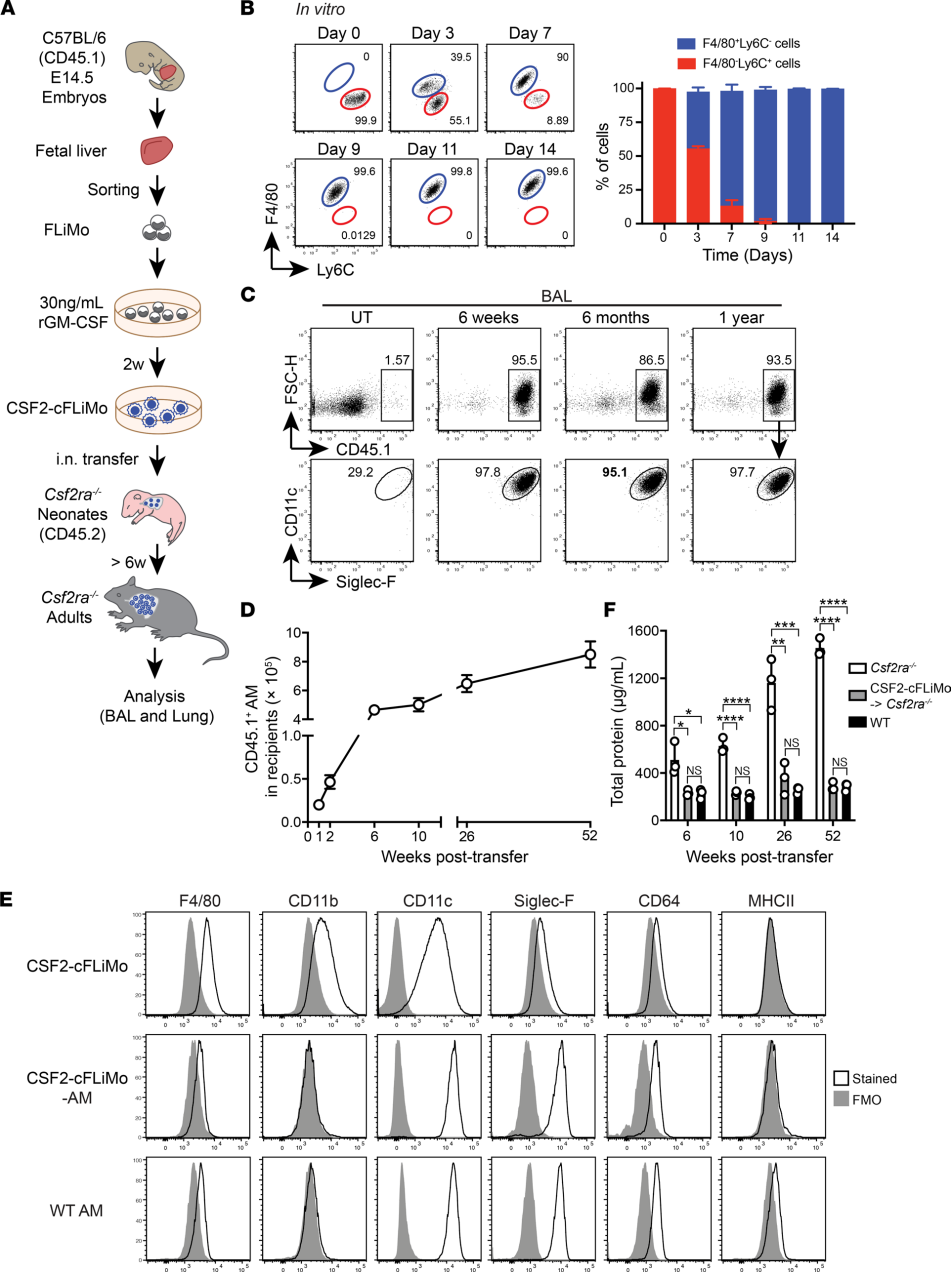
Fetal liver monocytes can proliferate in vitro with GM-CSF and further develop into mature functional alveolar macrophages in vivo. (**A**) Illustration of experimental scheme. (**B**) F4/80 and Ly6C expression on fetal liver monocytes cultured in vitro with GM-CSF (CSF2-cFLiMo) at the indicated time points. Shown are representative dot plots pre-gated on viable CD45^+^ single cells (left panel) and a column graph with percentages of F4/80^+^Ly6C^–^ and F4/80^–^Ly6C^+^ cells (right panel). (**C**–**F**) FLiMo were isolated from E14.5 embryos (CD45.1) and grown for 2 weeks with GM-SCF prior to i.n. transfer of 5 × 10^4^ cells to neonatal *Csf2ra^–/–^* (CD45.2) mice within the first 3 days after birth. (**C**) Shown are representative dot plots pre-gated on viable CD45.1 donor-derived cells from the bronchoalveolar lavage (BAL) of recipients (*n* = 3) at indicated time points after transfer. Untreated (UT) *Csf2ra^–/–^* recipient mice (6 weeks old) are included as a control (*n* = 3). (**D**) Total number of donor-derived alveolar macrophages (AMs) in the lung determined in recipients at the indicated time points (*n* = 3/time point). (**E**) Cell surface expression of AM markers on CSF2-cFLiMo prior to transfer and CSF2-cFLiMo–derived AMs 6 weeks after transfer to *Csf2ra^–/–^* neonates as well as endogenous AMs from age-matched control mice. Shown are representative histograms with the black line indicating specific staining and gray areas depicting fluorescence minus one controls (*n* = 3/group). (**F**) Total protein in the BAL of indicated groups of mice. The data are representative of 3 independent experiments. Data are presented as mean ± SD. ANOVA (1-way) was used in **F**. NS, not significant; *P < 0.05, **P < 0.01, ***P < 0.001, ****P < 0.0001.

The fetal liver contains several myeloid precursors, including primitive macrophages and erythro-myeloid progenitor-derived monocytes (20, 21). Recently, we and others have shown that the latter are the most potent precursors of alveolar macrophages (22, 23). To identify and better characterize the alveolar macrophage precursor, we sorted populations of viable F4/80^lo^CD11b^int^Ly6C^+^ monocytes and viable F4/80^hi^CD11b^lo^ primitive macrophages from the fetal liver of E14.5 C57BL/6 embryos (Supplemental Figure 1A; supplemental material available online with this article; https://doi.org/10.1172/jci.insight.152271DS1) and cultured them with GM-CSF in vitro (Figure 1A). Fetal liver monocytes proliferated vigorously in culture, indicated by Ki67 expression in 83%–98% cells, and accordingly, the number of cells grew exponentially (Supplemental Figure 1, B and C), whereas primitive macrophages disappeared within 2 weeks (Supplemental Figure 1B). At day 3 of the monocyte culture, a new population with macrophage characteristics (F4/80^+^Ly6C^–^ cells) emerged, which quickly predominated and appeared to be homogenous after 9 days in the culture (Figure 1B). The monocyte-to-macrophage differentiation was paralleled by an increase in cell granularity (SSC) and expression of the surface markers CD11c and Siglec-F (Supplemental Figure 1D). Similar phenotypic changes also occur during fetal monocyte to alveolar macrophage differentiation in the lung, as described previously (5, 6). Notably, the alveolar macrophage–like phenotype was maintained for at least 4 months of continuous culture (Supplemental Figure 1E). From here on, we refer to these F4/80^+^CD11c^+^CD11b^+^Siglec-F^+^ cells cultured with GM-CSF as CSF2-cFLiMo. Similar results were obtained when CSF2-cFLiMo were generated from fetal liver monocytes isolated from E16.5, E18.5, and E20.5 fetal livers (Supplemental Figure 1F). When differentiated CSF2-cFLiMo were deprived of GM-CSF, their numbers rapidly declined (Supplemental Figure 1, G and H), although the remaining cells maintained the expression of alveolar macrophage surface markers, including CD11c and Siglec-F (Supplemental Figure 1I). Notably, we found that addition of TGF-β increased the proliferative capacity and the yield of CSF2-cFLiMo (Supplemental Figure 2, A–D), while culture with TGF-β in the absence of GM-CSF failed to induce proliferation and resulted in the death of fetal liver monocytes (Supplemental Figure 2, B and C). Moreover, TGF-β did not change the phenotype of CSF2-cFLiMo after 2-week culture (Supplemental Figure 2D). These data indicated that TGF-β promoted expansion of alveolar macrophage precursors in the presence of GM-CSF, but was not required for their differentiation (24).

Taken together, fetal liver monocytes cultured with GM-CSF in vitro gave rise to a stable population of cells with alveolar macrophage–like phenotype and GM-CSF–dependent self-renewing capacity.

### CSF2-cFLiMo develop into mature and functional alveolar macrophages in vivo.

To assess whether CSF2-cFLiMo can develop into bona fide alveolar macrophages and perform alveolar macrophage function in vivo, we transferred congenically marked (CD45.1/CD45.2) CSF2-cFLiMo (i.n.) to newborn *Csf2ra^–/–^* mice (Figure 1A), which lack alveolar macrophages (9, 22). Analysis of the BAL and lung of *Csf2ra^–/–^* recipients showed efficient engraftment of donor-derived cells that resembled mature CD11c^hi^Siglec-F^hi^ alveolar macrophages (Figure 1C and Supplemental Figure 3A). The numbers of CSF2-cFLiMo–derived alveolar macrophages rapidly increased within the first 6 weeks after transfer before reaching a relatively stable population size (Figure 1D), similar to the kinetics during normal postnatal alveolar macrophage differentiation (5, 6). Three weeks after transfer, around 25% of CSF2-cFLiMo–derived alveolar macrophages were proliferating according to Ki67 positivity (Supplemental Figure 3B), while apoptotic cells were less than 10% (Supplemental Figure 3C), which is consistent with frequencies of proliferating and apoptotic alveolar macrophages found in 3-week-old naive WT mice (Supplemental Figure 3, B and C). Although CSF2-cFLiMo were CD11b^hi^Siglec-F^lo^ before transfer, they downregulated CD11b and upregulated CD11c and Siglec-F surface expression upon transfer and expansion in vivo, indicating that they completed their differentiation to become cells with a phenotype that was indistinguishable from alveolar macrophages of age-matched WT mice (Figure 1E). Notably, CSF2-cFLiMo–derived alveolar macrophages were maintained in the lung for at least 1 year after transfer (Figure 1, C and D). Moreover, measurement of protein concentration in the BAL at different time points after transfer showed that CSF2-cFLiMo-alveolar macrophage–reconstituted *Csf2ra^–/–^* mice were completely protected from PAP for up to 1 year (Figure 1F and Supplemental Figure 3D). These results demonstrated that CSF2-cFLiMo developed into mature alveolar macrophages, which appeared functionally equivalent to in situ differentiated alveolar macrophages.

### CSF2-cFLiMo–derived alveolar macrophages can self-renew in vivo.

**Figure 2:**
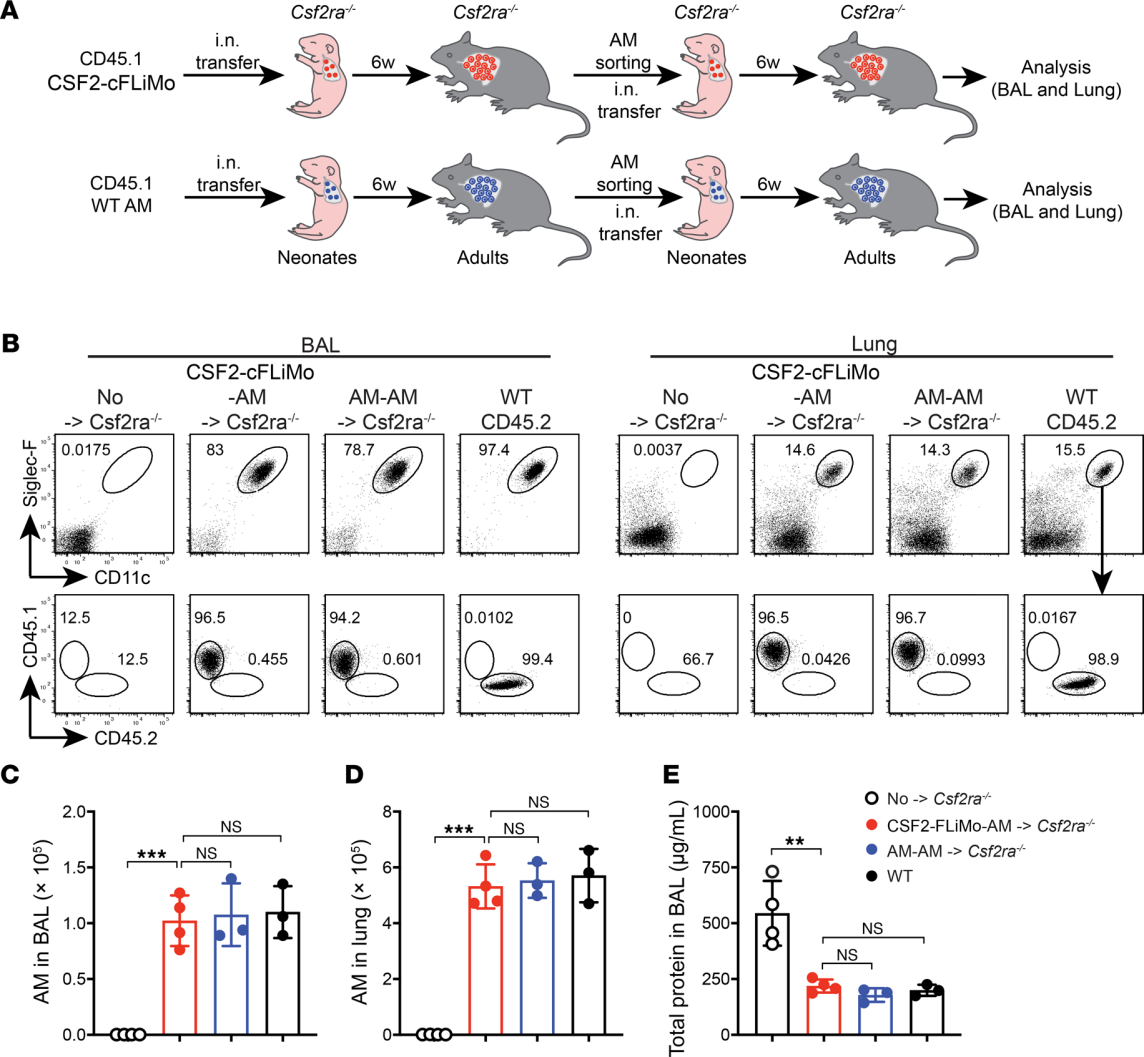
CSF2-cFLiMo–derived alveolar macrophages have the ability to self-renew in vivo. (**A**) Illustration of experimental regimen. CSF2-cFLiMo (CD45.1) or mature alveolar macrophages (AMs) isolated from adult mice were transferred i.n. to neonatal CD45.2 *Csf2ra^–/–^* mice. After 6 weeks, donor-derived AMs were sorted and 5 × 10^4^ of cells were transferred i.n. again to neonatal *Csf2ra^–/–^* mice. BAL and lung were analyzed 6 weeks after second-round transfer in **B**–**E**. (**B**) Representative dot plots showing the phenotype of CD45.1 donor-derived AMs in the BAL and lung, pre-gated as viable CD45^+^ single cells (*n* = 3 to 4/group). (**C** and **D**) Numbers of donor-derived AMs and WT AMs in the BAL (**C**) and lung (**D**) (*n* = 3 to 4/group). (**E**) Total protein in the BAL (*n* = 3 to 4/group). Age-matched *Csf2ra^–/–^* (*n* = 4) and CD45.2 WT (*n* = 3) mice were included as negative and positive controls, respectively. The data are representative of 3 experiments. Data are presented as mean ± SD. ANOVA (1-way) was used in **C**–**E**. NS, not significant; **P < 0.01, ***P < 0.001.

Alveolar macrophages are maintained locally through self-renewal, and they are largely independent of adult hematopoiesis at steady state (5, 6). Serial transplantation remains the gold standard for experimental assessment of long-term repopulating and self-renewal capacity of hematopoietic stem cells (25), but similar experiments have not been done for tissue-resident macrophages. To determine the long-term self-renewing capacity of CSF2-cFLiMo–derived alveolar macrophages, we serially transferred in vitro differentiated CSF2-cFLiMo or ex vivo isolated alveolar macrophages from adult WT mice into neonatal *Csf2ra^–/–^* mice. After 6 weeks, donor-derived alveolar macrophages were isolated and transferred into a second group of neonatal *Csf2ra^–/–^* recipients (Figure 2A). After secondary transfer, CSF2-cFLiMo–derived alveolar macrophages again fully restored the alveolar macrophage compartment of *Csf2ra^–/–^* recipients and their number and phenotype were comparable to alveolar macrophages derived from the second transfer of mature (alveolar macrophage–alveolar macrophage) and alveolar macrophages from unmanipulated WT mice (Figure 2, B–D). Furthermore, CSF2-cFLiMo–derived alveolar macrophages could successfully prevent proteinosis in *Csf2ra^–/–^* recipients after secondary transfer (Figure 2E). These results demonstrated that CSF2-cFLiMo–derived alveolar macrophages have a self-renewal capacity in vivo, which is comparable to bona fide alveolar macrophages.

### CSF2-cFLiMo acquire alveolar macrophage–specific transcriptional signature in vivo.

**Figure 3:**
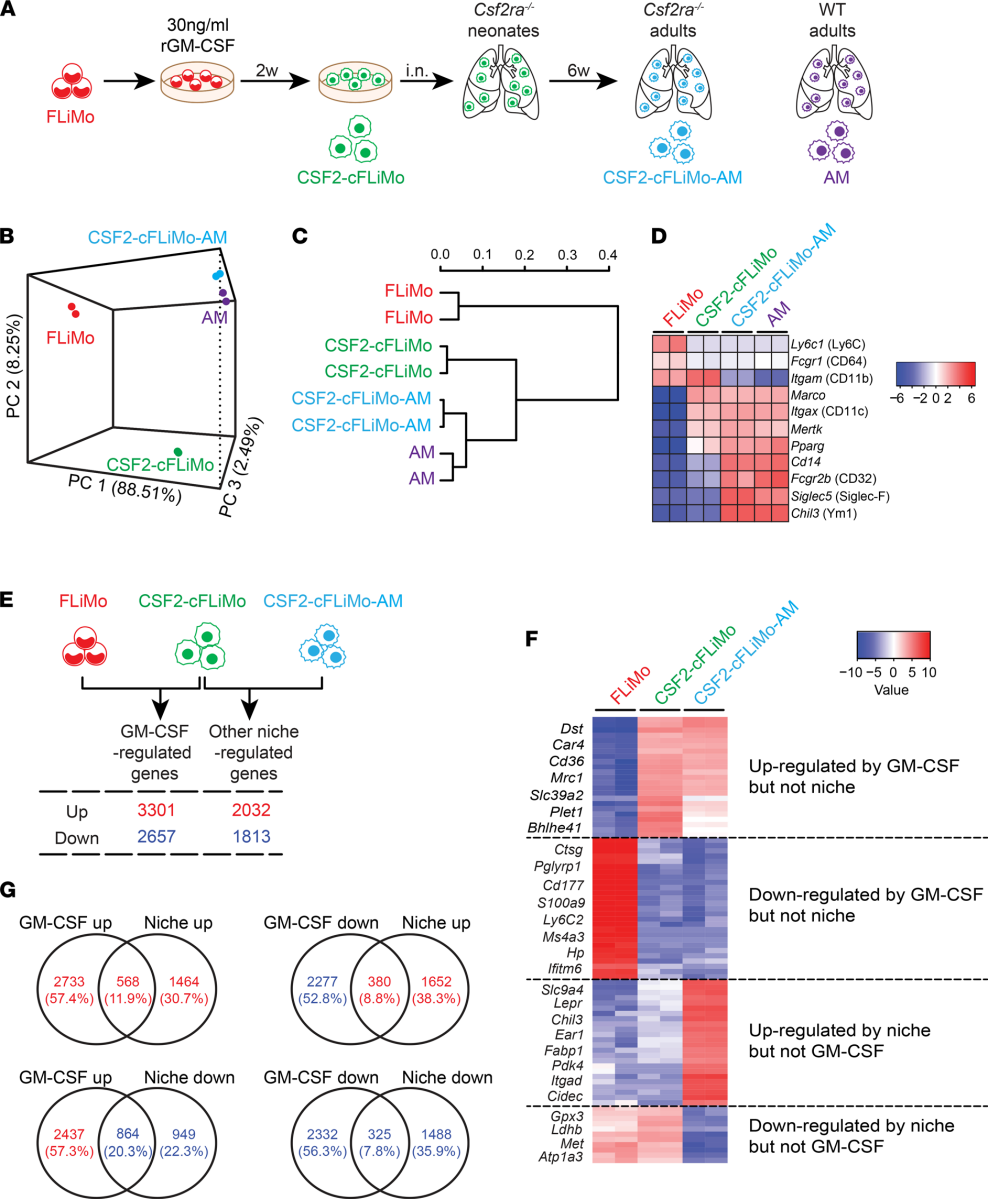
Gene expression profiles of transferred CSF2-cFLiMo in *Csf2ra^–/–^* mice. (**A**) Illustration of experimental regimen. Primary fetal liver monocytes (FLiMo) from E14.5 embryos, CSF2-cFLiMo cultured 2 weeks prior to transfer, ex vivo CSF2-cFLiMo–derived mature alveolar macrophages (AMs) from *Csf2ra^–/–^* recipients 6 weeks after transfer, and AMs from adult naive mice were sorted using flow cytometry. RNA-Seq was performed (2 biological replicates per group). (**B**) Principal component analysis (PCA) and (**C**) matrix clustering of the transcriptomes of all samples are shown. (**D**) Heatmaps showing expression of monocyte and AM markers. (**E**) The numbers of upregulated and downregulated genes by CSF2 or niche. (**F**) Heatmap showing the top 100 differentially expressed genes and representative genes of CSF2 and niche regulated. (**G**) Venn diagram of differentially expressed genes. Intersections of CSF2-upregulated or CSF2-downregulated versus niche-upregulated or niche-downregulated genes. The absolute gene numbers and percentages in the intersections are shown.

To reveal the gene expression programs that are associated with particular alveolar macrophage differentiation stages, we compared the transcriptomes of (a) ex vivo fetal liver monocytes from E14.5 embryos, (b) cultured CSF2-cFLiMo prior to transfer, (c) ex vivo CSF2-cFLiMo–derived alveolar macrophages, and (d) alveolar macrophages from adult naive mice (Figure 3A). Sorting strategies for fetal liver monocytes, CSF2-cFLiMo, and alveolar macrophages are shown in Supplemental Figure 1A and Supplemental Figure 4A, respectively. Principal component analysis (PCA) and matrix clustering based on all detected genes revealed that transcriptomes of CSF2-cFLiMo–derived alveolar macrophages and bona fide alveolar macrophages were similar and clustered closely together, while their gene expression profiles differed substantially compared with CSF2-cFLiMo prior to transfer. Most distantly related was the fetal liver monocyte population (Figure 3, B and C). CSF2-cFLiMo–derived alveolar macrophages and bona fide alveolar macrophages expressed low levels of several well-known monocyte markers, including *Ly6c1* (Ly-6C), *Fcgr1* (CD64), and *Itgam* (CD11b) (Figure 3D). Moreover, the relative mRNA expression of several well-established alveolar macrophage markers, including *Marco*, *Pparg*, *Itgax* (CD11c), *Mertk*, *Cd14*, *Fcgr2b* (CD32), *Siglec5* (Siglec-F), and *Chil3* (Ym1) was similar between CSF2-cFLiMo-alveolar macrophages and ex vivo alveolar macrophages (Figure 3D). Next, we compared the published gene signatures of monocytes and alveolar macrophages (26) with differential gene expression in CSF2-cFLiMo versus ex vivo fetal liver monocytes and CSF2-cFLiMo–derived alveolar macrophages versus CSF2-cFLiMo prior to transfer. More than 60% of monocyte signature genes were downregulated and 60% of alveolar macrophage signature genes were upregulated in fetal liver monocytes cultured with GM-CSF in vitro (Supplemental Figure 4B), suggesting that these alveolar macrophage signature genes are directly or indirectly regulated by GM-CSF. Interestingly, the remaining 40% alveolar macrophage signature genes were upregulated in CSF2-cFLiMo upon transfer and maturation in vivo (Supplemental Figure 4C).

The in vitro culture of precursors followed by in vivo transfer described here creates a model to study GM-CSF and other niche factors during alveolar macrophage development. Comparison of the CSF2-cFLiMo and fetal liver monocyte transcriptomes revealed 3301 upregulated and 2657 downregulated genes, which were dependent on GM-CSF (Figure 3E). Similarly, comparing the transcriptomes of CSF2-cFLiMo–derived alveolar macrophages to CSF2-cFLiMo, we found 2032 genes that were upregulated and 1813 genes that were downregulated by niche factors (Figure 3E). The representative genes of the top 100 differentially expressed genes regulated by GM-CSF or niche factors are listed in Figure 3F. Only a minor fraction of GM-CSF upregulated genes was further upregulated (11.9%) or downregulated (20.3%) by niche factors (Figure 3G). Similarly, 7.8% and 8.8% of GM-CSF downregulated genes were further downregulated or upregulated by niche factors, respectively (Figure 3G). These results showed that the majority of genes were separately regulated by GM-CSF and additional niche factors. More than 60% of the gene expression changes were driven by GM-CSF (Figure 3G), indicating its major contribution to alveolar macrophage development.

Taken together, our results showed that culture of fetal liver monocytes with GM-CSF in vitro resulted in alveolar macrophage–like precursors, which can accomplish full alveolar macrophage differentiation driven by alveolar niche factors upon i.n. transfer to alveolar macrophage–deficient neonates.

### CSF2-cFLiMo–derived alveolar macrophages are functional in homeostasis.

**Figure 4:**
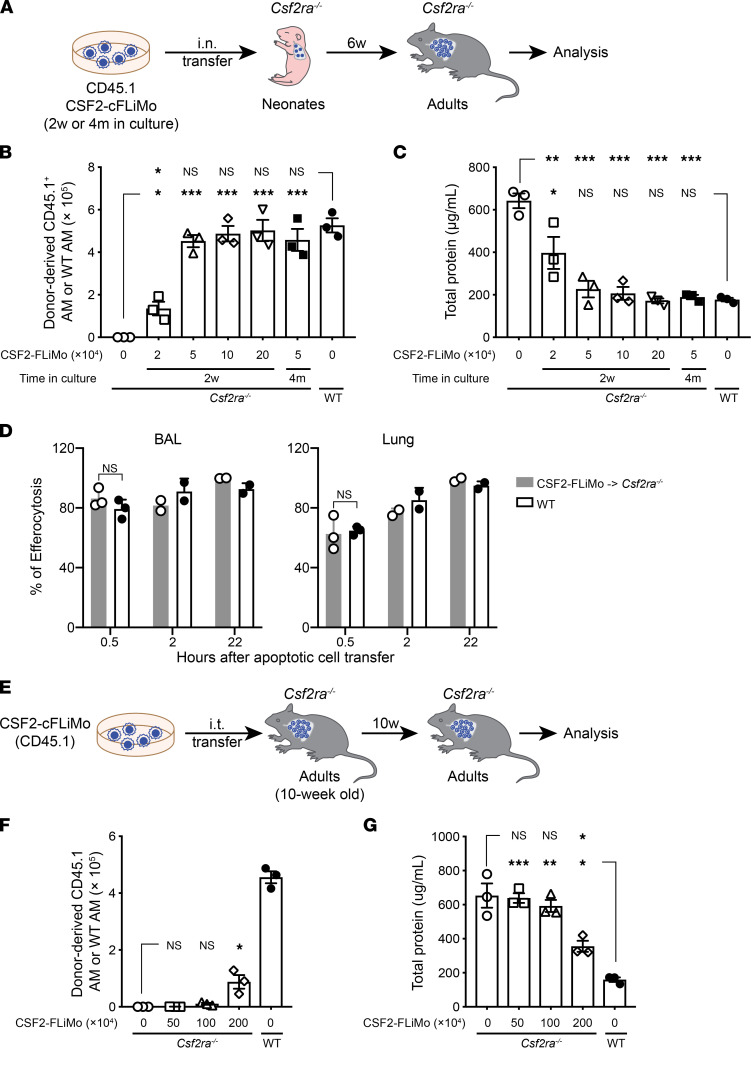
CSF2-cFLiMo–derived alveolar macrophages are functional in phagocytosis and efferocytosis. (**A**) Illustration of experimental regimen. Different numbers of CSF2-cFLiMo (CD45.1) after 2 weeks (2w) or 4 months (4m) of culturing were transferred i.n. to neonatal CD45.2 *Csf2ra^–/–^* mice and analyzed 6 weeks later in **B** and **C**. (**B**) Total numbers of donor-derived alveolar macrophages (AMs) in the lung of recipient *Csf2ra^–/–^* mice or WT mice (*n* = 3/group). (**C**) Total protein levels in the BAL (*n* = 3/ group). (**D**) Efferocytosis of i.t. instilled apoptotic thymocytes by AMs at the indicated time points. Values shown depict percentages of efferocytotic AMs (*n* = 2 to 3/group). (**E**) Illustration of experimental regimen. CD45.1 CSF2-cFLiMo were generated from E14.5 embryos and cultured 2 weeks in vitro. Different numbers of CSF2-cFLiMo were transferred i.t. to 10-week-old adult *Csf2ra^–/–^* mice and analyzed 10 weeks later in **F** and **G**. (**F**) Total numbers of donor-derived AMs in the BAL and lung of recipient *Csf2ra^–/–^* mice or AMs in the BAL and lung of WT mice (*n* = 3/group). (**G**) Total protein levels in the BAL. Age-matched *Csf2ra^–/–^* (*n* = 3) and WT (*n* = 3) mice were included as negative and positive controls, respectively. Data are presented as mean ± SD and the results are representative of 3 experiments. Student’s *t* test (unpaired) was used in **D** and ANOVA (1-way) was used in **B**, **C**, **F**, and **G**. NS, not significant; *P < 0.05, **P < 0.01, ***P < 0.001.

Overall, our studies demonstrated that CSF2-cFLiMo-alveolar macrophages were functionally equivalent to naturally differentiated alveolar macrophages. To determine the number of donor cells required to fully reconstitute the alveolar macrophage compartment of *Csf2ra^–/–^* mice, we titrated the number of transferred CSF2-cFLiMo (Figure 4A). Transfer of a minimum of 5 × 10^4^ CSF2-cFLiMo to neonatal *Csf2ra^–/–^* mice resulted in alveolar macrophage numbers in adult recipients that were comparable to unmanipulated WT mice (around 5 × 10^5^) (Figure 4B) and protected mice from PAP (Figure 4C). We have previously established that around 10% of primary fetal liver monocytes supplied i.n. reach the lung (22). Thus, CSF2-cFLiMo expanded around 100-fold 6 weeks after transfer to *Csf2ra^–/–^* neonates. Notably, extended time (i.e., 4 months) of CSF2-cFLiMo in vitro culture prior to transfer into recipient mice did not negatively affect their differentiation and functional capacity (Figure 4, B and C, and Supplemental Figure 5, A and B). Another critical function of tissue-resident macrophages, including alveolar macrophages, is the removal of apoptotic cells (efferocytosis) (27). We compared efferocytosis between CSF2-cFLiMo-alveolar macrophages in *Csf2ra^–/–^* mice and alveolar macrophages in WT mice by intratracheal (i.t.) instillation of labeled apoptotic thymocytes. CSF2-cFLiMo-alveolar macrophages and alveolar macrophages were equally potent at phagocytosing apoptotic cells from the bronchoalveolar space (Figure 4D). Furthermore, CSF2-cFLiMo–derived alveolar macrophages displayed comparable cytokine and chemokine genes with WT alveolar macrophages (Supplemental Figure 4D).

Next, we assessed whether CSF2-cFLiMo show therapeutic activity upon transfer into adult *Csf2ra^–/–^* mice, which had already developed PAP. Adult *Csf2ra^–/–^* mice (8–12 weeks) were transferred i.t. with 0.5, 1, or 2 million CSF2-cFLiMo (Figure 4, E–G). Ten weeks after transfer, donor-derived alveolar macrophages were detectable in the BAL and lung of *Csf2ra^–/–^* only in recipients transferred with 2 × 10^6^ cells (Figure 4F). The protein levels in the BAL from mice transferred with 2 × 10^6^ cells were significantly lower when compared with naive *Csf2ra^–/–^* mice, suggesting that transferred cells were able to reduce proteinosis, although not to the level of WT mice (Figure 4G). However, CSF2-cFLiMo–derived alveolar macrophages exhibited higher expression of F4/80 and CD11b and lower expression of Siglec-F and CD64 when compared with WT alveolar macrophages (Supplemental Figure 5, C and D), indicating that the alveolar macrophage phenotype was not fully recapitulated but intermediate between CSF2-cFLiMo and alveolar macrophages derived from CSF2-cFLiMo transferred to neonates. These results showed that CSF2-cFLiMo can reproduce alveolar macrophage phenotype and function most adequately when transferred to neonatal *Csf2ra^–/–^* mice.

### CSF2-cFLiMo–derived alveolar macrophages are superior to CSF1-cBMM–derived alveolar macrophages in reconstitution of empty alveolar macrophage niches and prevention of morbidity to infection with influenza virus.

**Figure 5:**
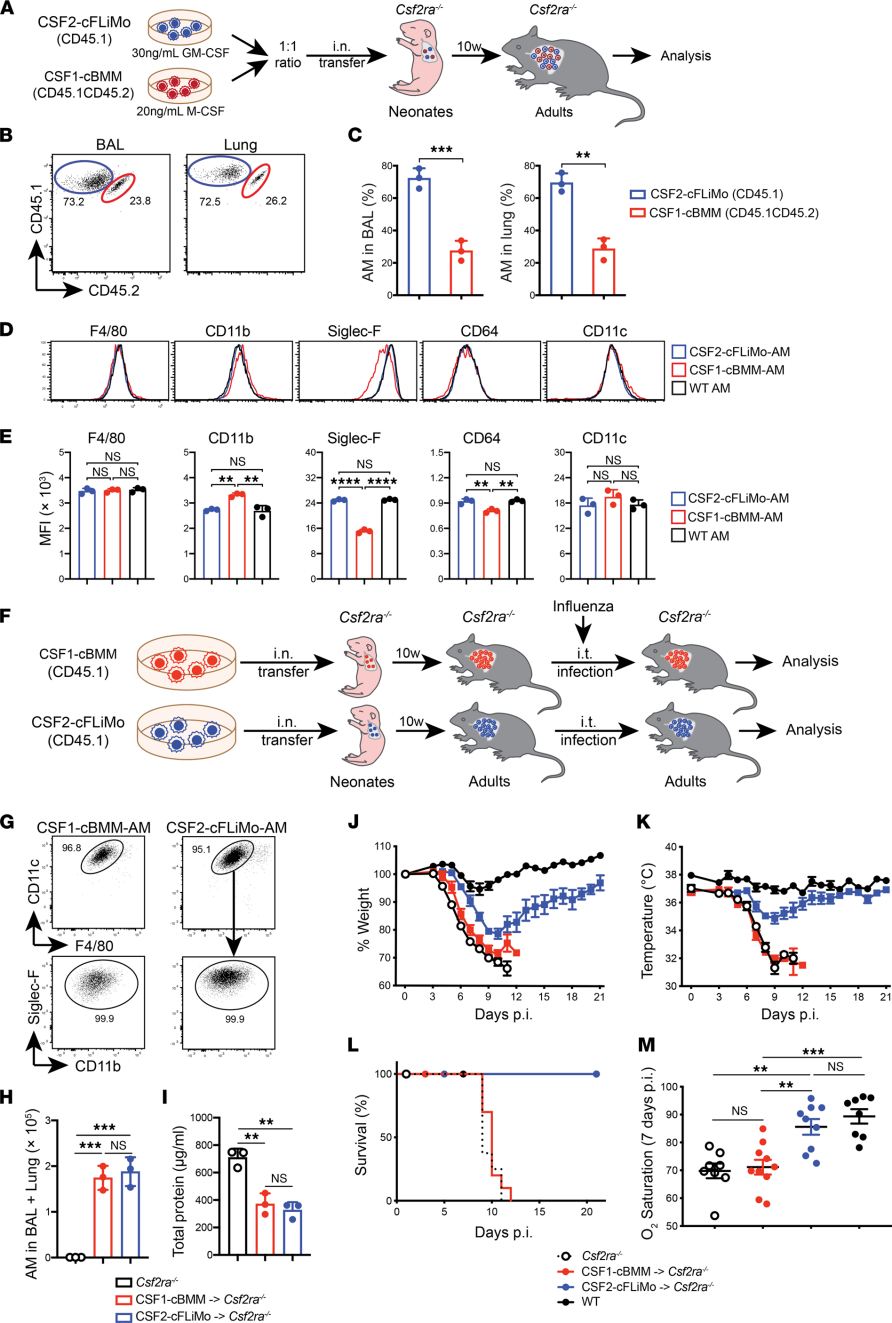
Influenza virus–induced disease severity is increased in *Csf2ra^–/–^* mice containing CSF1-cBMM–derived alveolar macrophages compared with *Csf2ra^–/–^* mice containing CSF2-cFLiMo. (**A**) Illustration of experimental regimen. Fetal liver monocytes were isolated from E14.5 embryos (CD45.1) and cultured 2 weeks in vitro to generate CSF2-cFLiMo. BM was isolated from adult mice (CD45.1CD45.2) and cultured 7 days with M-CSF in vitro to generate CSF1-cBMM. CSF2-cFLiMo and CSF1-cBMM were pooled in 1:1 ratio and transferred i.n. to neonatal *Csf2ra^–/–^* mice (CD45.2). (**B**–**E**) Recipients were analyzed 10 weeks later. (**B**) Representative dot plots and (**C**) percentages of donor-derived alveolar macrophages (AMs) in BAL and lung of the recipients (*n* = 3/group). (**D** and **E**) Shown are representative histograms (**D**) and MFI (**E**) of AM markers on CSF2-cFLiMo– and CSF1-cBMM–derived AMs in BAL of recipients, as well as AMs from BAL of untreated WT mice (*n* = 3/group). (**F**) Illustration of experimental scheme. *Csf2ra^–/–^* neonates (CD45.2) were transferred with CD45.1 CSF2-cFLiMo or CSF1-cBMM and analyzed in **G**–**M**. (**G**–**I**) The phenotype of donor-derived AMs in the BAL (pre-gated as viable CD45.1^+^ singlets) (**G**), the number of donor-derived AMs (**H**), and proteinosis in recipients (**I**) was determined 10 weeks after transfer prior infection (*n* = 3 to 4/group). (**J**–**M**) Mice were infected with 10 PFU influenza virus (PR8) and morbidity was analyzed at indicated days (*n* = 9–10/group); p.i., post-infection. Shown are percentage body weight relative to the day of infection (**J**), body temperature (**K**), survival curve (**L**), and O_2_ saturation at 7 days after infection (**M**). Age-matched *Csf2ra^–/–^* and WT mice were included as negative and positive controls (*n* = 8/group). Data are presented as mean ± SD and the results are representative of 3 experiments. Student’s *t* test (unpaired) was used in **C** and ANOVA (1-way) was used in **E**, **H**, **I**, and **M**. NS, not significant; **P < 0.01, ***P < 0.001, ****P < 0.0001.

Recent studies have shown that transplantation of in vitro–generated BMMs could decrease proteinosis in adult *Csf2rb^–/–^* mice in homeostasis (15, 16). To directly compare the capacity of CSF1-cBMM and CSF2-cFLiMo in alveolar macrophage development, we transferred both populations at a 1:1 ratio into neonatal *Csf2ra^–/–^* recipients (Figure 5A). Analysis of the alveolar macrophage compartment 10 weeks after transfer showed that around 70% of alveolar macrophages were derived from CSF2-cFLiMo (Figure 5, B and C) and that their phenotype (CD11b^lo^Siglec-F^hi^) closely resembled the phenotype of genuine alveolar macrophages, while the remaining 30% CSF1-cBMM–derived alveolar macrophages showed increased CD11b and a reduction of Siglec-F and CD64 on the cell surface and in RNA levels (Figure 5, D and E, and Supplemental Figure 6). Otherwise, no difference was seen in the expression of alveolar macrophage signature genes (26), including *Pparg*, *Ear1*, *Cidec*, *Krt19*, *Trim29*, *Clmn*, *Atp10a*, *Mfsd7c*, and *Slc6a4,* when comparing CSF1-cBMM–derived and CSF2-cFLiMo–derived alveolar macrophages (Supplemental Figure 6).

Importantly, similar results were obtained by transfer of 1:1 mixtures of CSF2-cFLiMo and CSF2-cBMM (Supplemental Figure 7, A–C) or CSF2-cFLiMo mixed with BMMs derived from cultures containing both M-CSF and GM-CSF (CSF1+CSF2-cBMM) (Supplemental Figure 7, D–F), demonstrating that the impaired efficiency of CSF1-cBMMs in reconstitution of empty alveolar macrophage niches was not due to the absence of CSF2 in vitro.

In addition to the homeostatic function, alveolar macrophages play an essential role in protecting influenza virus–infected mice from morbidity by maintaining lung integrity through the removal of dead cells and excess surfactant (6). To assess the functional capacity of CSF2-cFLiMo–derived alveolar macrophages during pulmonary virus infection, we reconstituted *Csf2ra^–/–^* neonates with CSF2-cFLiMo and infected adults 10 weeks later with influenza virus PR8 (Figure 5, F–M). *Csf2ra^–/–^* mice lacking alveolar macrophages succumbed to infection due to lung failure (Figure 5, J–M) as reported previously (9). Notably, the presence of CSF2-cFLiMo–derived alveolar macrophages protected *Csf2ra^–/–^* mice from severe morbidity (Figure 5, J and K) and completely restored viability (Figure 5L) and O_2_ saturation (Figure 5M) compared with infected WT mice.

Next, we reconstituted *Csf2ra^–/–^* mice with CSF2-cFLiMo or CSF1-cBMM (Figure 5, F–M) to compare their ability to ameliorate influenza-induced morbidity. Because of impaired capacity of CSF1-cBMMs to reconstitute alveolar macrophage niches of Csf2ra*^–/–^* mice (Figure 5B), we had to transfer 3 times more of them to guarantee that the number of mature alveolar macrophages and proteinosis in the BAL was comparable in the 2 groups (Figure 5, G–I). Upon influenza infection, *Csf2ra^–/–^* mice containing CSF1-cBMM–derived alveolar macrophages showed strikingly increased morbidity (i.e., loss of body weight and temperature; O_2_ saturation) and mortality compared with mice containing CSF2-cFLiMo–derived alveolar macrophages (Figure 5, J–M). These results demonstrated that reconstitution of alveolar macrophage–deficient mice with in vitro–generated BMMs was detrimental for the outcome of respiratory viral infection.

### Major and minor histocompatibility differences in transplanted CSF2-cFLiMo result in transplant rejection.

**Figure 6:**
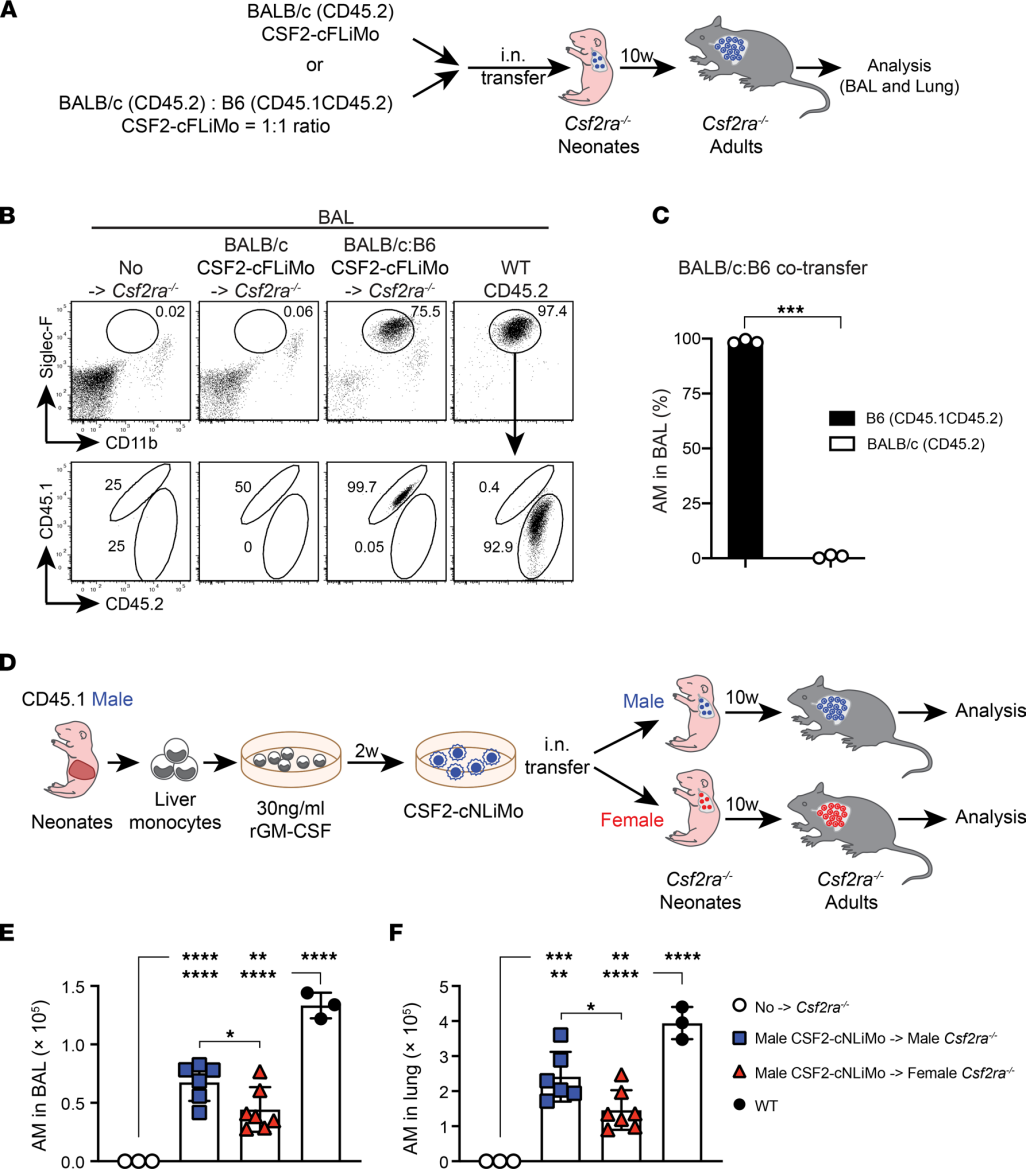
Rejection of allogenic CSF2-cFLiMo–derived alveolar macrophages. (**A**) Illustration of experimental regimen. CD45.2 BALB/c and CD45.1 B6 CSF2-cFLiMo were generated from E14.5 embryos and cultured 2 weeks in vitro. BALB/c CSF2-cFLiMo were transferred to neonatal CD45.2 *Csf2ra^–/–^* mice (B6 background) either separately or in a 1:1 ratio with B6 CSF2-cFLiMo and analyzed 10 weeks later in **B** and **C**. (**B**) Representative dot plots showing the phenotype of donor-derived alveolar macrophages (AMs) in the BAL, pre-gated on viable CD45^+^ single cells (*n* = 3/group). (**C**) Percentage of donor-derived AMs in BAL of cotransferred recipients (*n* = 3). (**D**) Illustration of experimental regimen. GM-CSF cultured neonatal liver monocytes (CSF2-cNLiMo) generated from CD45.1 WT male neonates after 2-week culture were i.n. transferred to neonatal CD45.2 *Csf2ra^–/–^* mice and analyzed after 10 weeks in **E** and **F**. Mice were grouped according to sex (*n* = 6 to 7/group). (**E** and **F**) Numbers of donor-derived and WT AMs in the BAL (**E**) and lung (**F**) are shown. Age-matched *Csf2ra^–/–^* and CD45.2 WT mice were included as negative and positive controls (*n* = 3/group). Data are presented as mean ± SD and the results are representative of 3 experiments. Student’s *t* test (unpaired) was used in **C** and ANOVA (1-way) was used in **E** and **F**. *P < 0.05, **P < 0.01, ***P < 0.001, ****P < 0.0001.

Our results suggest that CSF2-cFLiMo could be used as an elegant in vitro and in vivo system to study the development and function of alveolar macrophages. To gain more information about the potential of this system, we next evaluated the MHC compatibility in an allogeneic CSF2-cFLiMo transfer. We generated CSF2-cFLiMo from BALB/c E14.5 embryos and transferred them alone or together with CSF2-cFLiMo from C57BL/6 embryos at a 1:1 ratio into neonatal *Csf2ra^–/–^* mice on the C57BL/6 background (Figure 6A). Ten weeks later, we were unable to detect any BALB/c CSF2-cFLiMo–derived alveolar macrophages, irrespective of whether they were transferred alone or together with C57BL/6 CSF2-cFLiMo, indicating complete rejection of MHC-mismatched cells (Figure 6, B and C, and Supplemental Figure 8, A and B).

We then also compared Y chromosome compatibility by transferring GM-CSF–cultured monocytes isolated from the liver of male neonates (CSF2-cNLiMo) to *Csf2ra^–/–^* male or female neonatal recipients (Figure 6D). Ten weeks later, lungs of female recipients contained fewer mature alveolar macrophages derived from male donor cells than lungs of male recipients (Figure 6, E and F). In contrast, transfer of female donor cells to male and female *Csf2ra^–/–^* neonates resulted in a comparable number of mature alveolar macrophages (Supplemental Figure 8, C–E). Importantly, male and female CSF2-cNLiMo showed similar growth in vitro (Supplemental Figure 8F). These results indicate that proteins that are uniquely encoded on the Y chromosome and expressed by alveolar macrophages are sufficient for partial rejection in females.

Taken together, our data indicated that both major histocompatibility complex and minor histocompatibility antigens should be considered to avoid rejection of alveolar macrophage grafts.

### Gene therapy of Csf2ra-deficient fetal liver monocytes.

**Figure 7:**
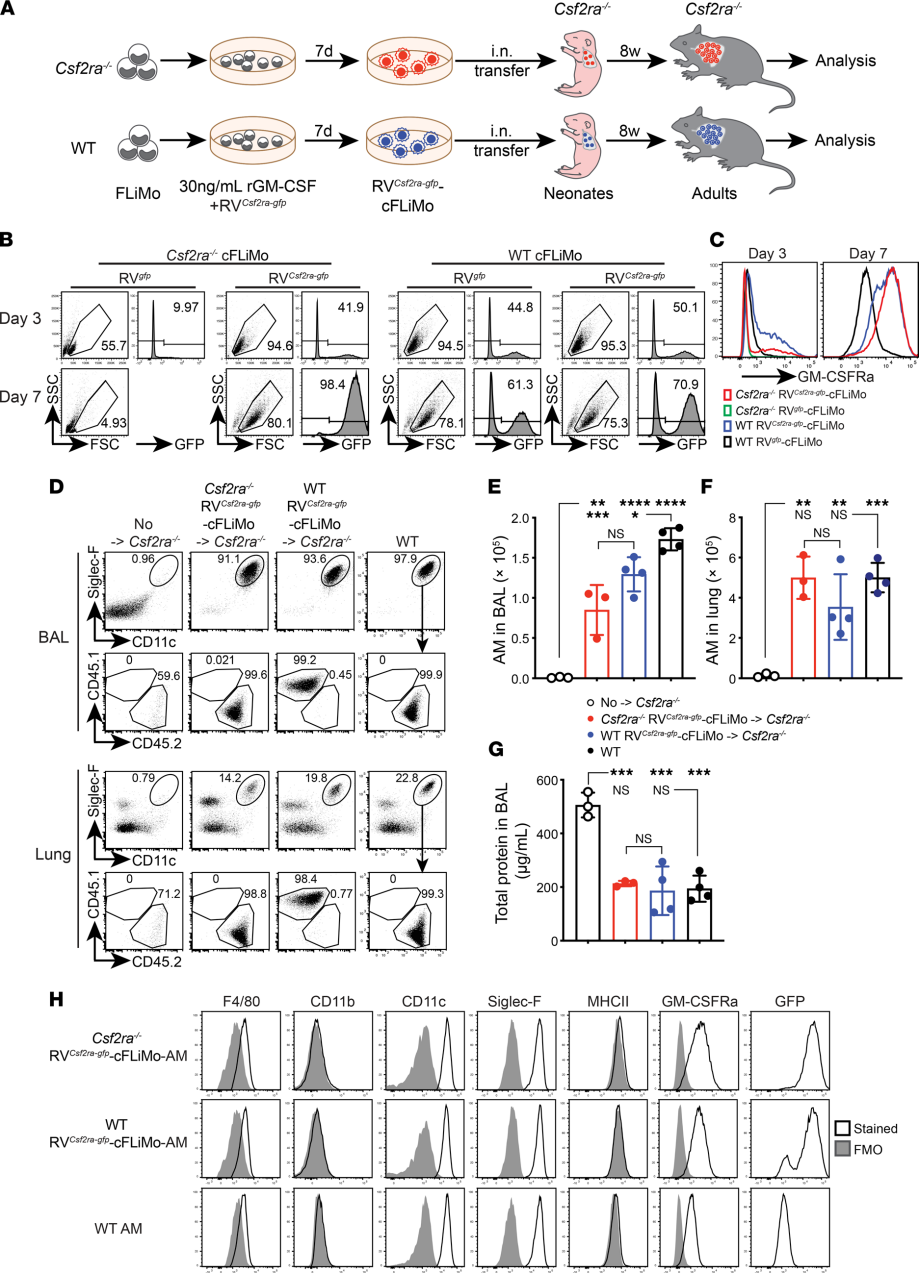
Gene therapy of *Csf2ra* deficiency by gene transfer to CSF2-cFLiMo. (**A**) Illustration of experimental regimen. Fetal liver monocytes (FLiMo) were purified from E14.5 CD45.2 *Csf2ra^–/–^* or CD45.1 WT embryos and spin-infected with a retrovirus encoding for Csf2ra and GFP (RV*^Csf2ra-gfp^*) or GFP alone (control RV*^gfp^*). Cells were cultured with CSF2 for 7 days. RV*^Csf2ra-gfp^-*transduced *Csf2ra^–/–^* CSF2-cFLiMo (RV*^Csf2ra-gfp^*-FLiMo) or identically treated CD45.1 WT CSF2-cFLiMo were transferred i.n. to neonatal CD45.2^+^ *Csf2ra^–/–^* mice and evaluated after 8 weeks. (**B**) Efficiency of spin infection (GFP^+^) and survival of cultured cells at days 3 and 7 after infection. (**C**) GM-CSF receptor alpha protein expression levels on the cell surface of *Csf2ra^–/–^* and WT FLiMo 3 and 7 days after transduction with RV*^Csf2ra-gfp^* or RV*^gfp^*, respectively. (**D**) Representative dot plots showing the phenotype of donor-derived cells in the BAL and lung, pre-gated on viable CD45^+^ single cells. (**E** and **F**) Numbers of donor-derived alveolar macrophages (AMs) and WT AMs in the BAL (**E**) and lung (**F**). (**G**) Total protein levels in the BAL. (**D**–**G**) Age-matched *Csf2ra^–/–^* and CD45.2 WT mice were included as negative and positive controls. (**H**) Representative histograms showing cell surface expression of characteristic proteins on AMs harvested from GM-CSFRa and GFP on RV*^Csf2ra-gfp^*-FLiMo–derived AMs and WT AMs showing fluorescence minus one control (gray) and specific antibodies against indicated markers (black line). (**B**–**H**) *n* = 3–4/group. Data are presented as mean ± SD and the results are representative of 3 experiments. ANOVA (1-way) was used in **E**–**G**. NS, not significant; *P < 0.05, **P < 0.01, ***P < 0.001, ****P < 0.0001.

The reconstitution of alveolar macrophages by transferring cultivatable precursors provides unique opportunities for genetic manipulation. To provide proof of concept, fetal liver monocytes were purified from E14.5 *Csf2ra^–/–^* or WT embryos and transduced with a retrovirus encoding *Csf2ra-gfp* (RV*^Csf2ra-gfp^*) or control *gfp* only (RV*^gfp^*) (Figure 7A). RV*^Csf2ra-gfp^*-transduced *Csf2ra-*deficient fetal liver monocytes expanded in the presence of GM-CSF in vitro (Figure 7B) and outgrew all nontransduced cells, as indicated by the presence of almost 100% GFP^+^ cells by day 7 of culture (Figure 7, B and C). As expected, *Csf2ra-*deficient fetal liver monocytes transduced with control RV*^gfp^* could not expand in culture (Figure 7, B and C). Notably, overexpression of *Csf2ra* in WT fetal liver monocytes also provided a slight growth advantage over nontransduced WT fetal liver monocytes in vitro (Figure 7, B and C). Next, we transferred RV*^Csf2ra-gfp^*-transduced *Csf2ra*-deficient fetal liver monocytes into neonatal *Csf2ra^–/–^* mice and analyzed the BAL and lung 8 weeks later. Indeed, gene therapy of *Csf2ra* deficiency in fetal liver monocytes enabled full reconstitution of a functional alveolar macrophage compartment that prevented development of PAP in *Csf2ra^–/–^* mice (Figure 7, D–G). Transfer of *Csf2ra*-overexpressing WT fetal liver monocytes did not result in higher alveolar macrophage numbers, indicating that GM-CSF bioavailability rather than receptor expression was the limiting factor. RV*^Csf2ra-gfp^*-transduced fetal liver monocyte–derived alveolar macrophages were phenotypically indistinguishable from unmanipulated WT alveolar macrophages for multiple surface markers, including F4/80, CD11b, CD11c, Siglec-F, and MHCII (Figure 7H). These results provide a proof of concept that gene-modified CSF2-cFLiMo differentiate into functional bona fide alveolar macrophages, therefore allowing genetic manipulation of this important tissue macrophage compartment.

## Discussion

We and others have previously shown that transfer of WT fetal liver or fetal lung monocytes can restore defective alveolar macrophage development in Csf2ra-deficient or Csf2rb-deficient mice (5, 6, 9, 22, 23). In the present study, we describe a 2-step alveolar macrophage differentiation model. In a first step, a homogenous and stable immature alveolar macrophage–like population (i.e., CSF2-cFLiMo) is generated from mouse fetal liver monocytes that can be maintained long-term in culture in the presence of GM-CSF. In a second step, upon pulmonary transplantation, CSF2-cFLiMo expand and complete differentiation to bona fide alveolar macrophages. Indeed, low numbers of CSF2-cFLiMo were sufficient to completely restore the alveolar macrophage pool of Csf2ra-deficient mice within 6 weeks. CSF2-cFLiMo–derived alveolar macrophages were stably maintained for at least 1 year, indicating that they efficiently occupied empty alveolar macrophage niches and possessed a long-term self-renewing capacity, which was proven by serial transplantation. While serial transplantation is the gold standard for proof of long-term repopulation and self-renewal capacity of hematopoietic stem cells, according to our knowledge, to date it has not been applied to tissue-resident macrophages.

Studies in knockout mice have shown that GM-CSF and TGF-β are essential for alveolar macrophage development, mainly by induction of the transcription factor PPARγ and its target genes (5, 6, 24). Our fetal liver monocyte cultures suggest that GM-CSF is sufficient for alveolar macrophage growth and differentiation of alveolar macrophage precursors, while TGF-β further promotes their proliferative capacity. TGF-β alone was insufficient to grow fetal liver monocytes in vitro. Notably, it has been suggested that fetal liver cells can be grown long-term in the presence of GM-CSF in vitro (28). However, in contrast to fetal liver monocytes, fetal liver macrophages, which are derived from yolk sac primitive macrophages, could not be expanded in vitro, despite the presence of Csf2ra and Csf2rb chains, consistent with their poor capacity to restore the alveolar macrophage compartment upon neonatal transfer to Csf2ra-deficient mice (22).

Even though fetal liver monocytes upregulated a large panel of alveolar macrophage signature genes in the presence of GM-CSF in vitro, PCA analysis of the transcriptome showed a difference compared with mature genuine alveolar macrophages. However, upon pulmonary transfer, CSF2-cFLiMo completed differentiation into bona fide alveolar macrophages, indicating that lung tissue instructs terminal differentiation of alveolar macrophages. Thus, the 2-step model allowed us to separate the transcriptional regulation induced by GM-CSF from other niche factors provided by the lung microenvironment during alveolar macrophage development. Further studies using this model will help to understand transcriptional regulation during alveolar macrophage development.

Notably, when CSF2-cFLiMo were transplanted into the lung of adult C*sf2ra*-deficient recipients, Siglec-F upregulation and CD11b downregulation occurred less efficiently as compared with pulmonary transplantation to neonates, indicating that CSF2-cFLiMo transplantation was more efficient in neonates than in adults. We can think of 2 possible reasons. Firstly, the occurrence of proteinosis in adult *Csf2ra^–/–^* mice might obstruct the contact between transplanted cells and lung epithelial cells, which is important for the survival and engraftment of transplanted cells. Secondly, the neonatal lung niche could more efficiently promote alveolar macrophage maturation compared with the adult lung niche. Thus, the macrophage transplantation treatment for patients with hereditary PAP would be more efficient during the neonatal or childhood stage before development of severe proteinosis. Importantly, we showed that the lung alveolus is not an immune-privileged site, indicating that transplantation of alveolar macrophage–like precursors to MHC- and even gender-mismatched recipients results in rejection. Thus, our data indicate that both MHC and Y chromosome compatibility require consideration when transferring CSF2-cFLiMo precursors.

M-CSF–derived BMMs have been widely used to study macrophage biology in vitro, although they poorly recapitulate the heterogenous phenotypic and functional features of genuine resident macrophage subsets that are present in every tissue. Despite this, M-CSF–derived BMMs or GM-CSF–derived BMMs can differentiate to alveolar macrophages upon pulmonary transfer to *Csf2rb-*deficient mice (15, 16). Furthermore, induced pluripotent stem cell–derived macrophages originating from primitive macrophages in the mouse yolk sac or from human blood CD34^+^ cells have been proposed as models for pulmonary macrophage transfer therapies (17–19). So far, the capacity to prevent alveolar proteinosis has served as the only functional parameter for quality control in all of these approaches.

Alveolar macrophages develop from fetal monocytes independent of yolk sac primitive macrophages and BM precursors in the steady state (7, 20). Several studies showed recently that alveolar macrophages derived from yolk sac primitive macrophages or from BM monocytes are different in metabolism and function (29–32). In fact, CSF2-cBMMs possess impaired capacity to engraft, expand, and acquire a bona fide alveolar macrophage phenotype in empty niches compared with CSF2-cFLiMo. These results demonstrated that catabolism of accumulated proteins in the BAL was insufficient to assess the efficacy of pulmonary macrophage transplantation therapies and that GM-CSF–cultured fetal liver monocytes were superior to alveolar macrophage–like cells derived from BM or blood cultures. CSF2-cFLiMo–derived alveolar macrophages prevented PAP, performed efferocytosis of apoptotic cells, and protected from fatal respiratory viral infection, indicating that they acquired the broad functional spectrum of bona fide alveolar macrophages.

CSF2-cFLiMo generated from WT or gene-deficient mice could be used as a high-throughput screening system to study alveolar macrophage development in vitro and in vivo. Our model is suitable to study the relationship between alveolar macrophages and lung tissue, as well as the roles of specific genes or factors in alveolar macrophage development and function. Furthermore, CSF2-cFLiMo can overcome the limitation in macrophage precursor numbers and be used as a therapeutic approach for PAP disease or in other macrophage-based cell therapies, including lung emphysema, lung fibrosis, lung infectious disease, and lung cancer (33–35). Indeed, macrophage-based therapies have been proposed for various diseases (36–39). Although the availability of human fetal liver may be limited, it should be noted that fetal liver biopsies have been successfully used for prenatal diagnosis of ornithine transcarbamylase deficiency, von Gierke’s disease, and primary hyperoxaluria type 1 between weeks 20 and 23 after gestation (40–43). Finally, genetically modified and transferred CSF2-cFLiMo might facilitate the controlled expression of specific therapeutic proteins in the lung for disease treatment, and therefore, could represent an attractive alternative to nonspecific gene delivery by viral vectors.

## Methods

### Mice.

C57BL/6 (strain 000664), C57BL/6 congenic (CD45.1) (strain 002014), and BALB/c (strain 000651) mice were originally from The Jackson Laboratory. *Csf2ra^–/–^* mice were established in our laboratory (9). All mice were housed and bred under specific pathogen–free conditions in individually ventilated cages in a controlled day-night cycle at the ETH Phenomics Facility. Mice used for experiments were 6–12 weeks of age (adults), unless otherwise stated.

### Timed pregnancy.

Female C57BL/6 CD45.1, CD45.2, or BALB/c mice were housed together with matching male mice overnight. The vaginal plug was checked on the next day and was designated as E0.5.

### Pulmonary cell transplantation.

Neonatal (days 0–3 after birth) *Csf2ra^–/–^* recipient mice were transferred i.n. with different numbers of cells in 10 μL endotoxin-free PBS. For competitive transfer experiments, 25,000–50,000 cells from each origin were mixed and transferred. Adult (8–12 weeks) *Csf2ra^–/–^* recipient mice were transferred i.t. with different numbers of cells in 50 μL endotoxin-free PBS.

### Cell suspension preparation.

Mice were euthanized by overdose (400 mg/kg body weight) of sodium pentobarbital by i.p. injection. The lungs were washed 3 times with 0.4 mL of ice-cold PBS containing 2 mM EDTA through an i.t. cannula only when mice were older than 4 weeks. BAL fluid was collected and cells were harvested by centrifugation. After collecting BAL fluid, ice-cold PBS was perfused through the right ventricle. The lungs were dissected, and other tissues and bronchi were removed. Pregnant females were euthanized by CO_2_ asphyxiation. Fetal livers were removed at the indicated time points. Organs were minced and then digested at 37°C in IMDM containing 2.0 mg/mL of type IV collagenase (Worthington Biochemical), 0.125 mg/mL DNase I (Sigma-Aldrich), and 3% FCS for 45 minutes (lungs) or 25 minutes (fetal and neonatal livers), respectively, and subsequently passed through a 70 μm cell strainer (Becton Dickinson). Ammonium-chloride-potassium (ACK) lysing buffer was used for erythrocyte lysis for all samples.

### Flow cytometry.

Multiparameter assessment was performed using LSR Fortessa (BD Biosciences) and data were analyzed with FlowJo software (TreeStar). After blocking the FcgIII/II receptors by incubation with homemade anti-CD16/32 (2.4G2), single-cell suspensions were incubated with the indicated fluorochrome-conjugated or biotinylated monoclonal antibodies in FACS buffer (PBS containing 2% FCS and 2 mM EDTA) and then washed twice before detection. Monoclonal antibodies specific to mice are listed in Table 1. Dead cells were excluded using the live/dead marker eFluor780 (Invitrogen).

### Cell counting.

Cell suspension for the BAL and lung samples was resuspended in 0.25 mL and 1 mL of FACS buffer (PBS containing 2% FCS and 2 mM EDTA), respectively. Next, 10 μL of samples was transferred to 96-well plates, and then diluted with 90 μL of FACS buffer. Total events were counted by taking 50 μL of diluted samples by flow cytometry. Then, cell numbers for different populations were calculated according to percentages of each population in the samples.

### Cell sorting.

Multiparameter cell sorting was performed using FACSAria III (BD Biosciences). After blocking the FcgIII/II receptors by incubation with homemade anti-CD16/32 (2.4G2), single-cell suspensions were incubated with the indicated fluorochrome-conjugated antibodies (see antibody list) in FACS buffer (PBS containing 2% FCS and 2 mM EDTA) and then washed twice before sorting. The fluorochrome signal was detected using an optical excitation laser and BP emission filter (optical sorter configuration shown in Table 2); a 100 μm nozzle and 2-way sorting mode were used. Purified cell samples were collected into FCS-coated 15 mL Falcon tubes and reanalyzed for purity after FACS sorting. Dead cells were excluded using the live/dead marker Sytox red (Invitrogen). Purity was always more than 95%, and cells were almost 100% alive. Gating hierarchy strategies are available in the supplemental material.

### Isolation and generation of CSF2-cFLiMo.

CSF2-cFLiMo were routinely prepared from fetal liver of E13.5 to E17.5 mouse embryos. In some experiments, CSF2-cFLiMo were prepared from fetal liver of E18.5, E20.5, or neonatal liver within the first 2 days after birth. Generally, all embryos from a litter were pooled. Male and female neonates were separately used and transferred to address minor histocompatibility antigen rejection. Fetal livers were minced and then digested at 37°C in IMDM containing 2.0 mg/mL of type IV collagenase (Worthington Biochemical), 0.125 mg/mL DNase I (Sigma-Aldrich), and 3% FCS for 15 minutes (for E13.5–E17.5 livers) or 30 minutes (for E18.5–neonatal livers) and subsequently passed through a 70 μm cell strainer (Becton Dickinson). RBCs were removed by ACK lysing buffer. Fetal liver monocytes were sorted using flow cytometry. Sorted cells were then seeded at a density of 1 × 10^5^ cells/mL in untreated 24-well plates (Sarstedt, 83.3922.500) or 12-well plates (Sarstedt, 83.3921.500) and maintained in GlutaMAX supplement RPMI 1640 (Gibco, 61870036), containing 10% FCS, 10 mM HEPES, 50 μM 2-mercaptoethanol, 100 U/mL penicillin, 100 U/mL streptomycin in the presence of 30 ng/mL murine GM-CSF (PeproTech, 315-03) in vitro. Cells were subcultured when cell density reached 5 × 10^5^ cells/mL, normally every 3–4 days. For this purpose, cells were detached with 4 mM EDTA in PBS, and then centrifuged for 5 minutes at 300*g* and resuspended in fresh medium. In some experiments, human TGF-β (PeproTech, 100-21c) was used for culture with or without GM-CSF. CSF2-cFLiMo used for transplantation were cultured for 2 weeks in culture media prior to transfers unless stated otherwise.

### Generation of BMMs.

For the preparation of mouse BMMs, tibias and femurs from the hind legs of adult (6–12 weeks old) donor mice were flushed with PBS. BM was rinsed through a 70 μm cell strainer (Becton Dickinson) followed by RBC depletion with ACK lysing buffer. BMMs were differentiated in vitro in complete RPMI, supplemented with 20 ng/mL M-CSF (PeproTech, 315-02) and/or 30 ng/mL GM-CSF (PeproTech, 315-03). Medium was replaced on day 3 and day 6. Adherent cells were harvested and used as mature BMMs on day 7.

### Assessment of total protein.

Total protein concentrations in BAL fluid were detected by Pierce BCA protein assay kit (Thermo Fisher Scientific, 23227) according to the manufacturer’s instructions.

### RNA-Seq.

First, 100,000 cells of indicated populations were collected into TRIzol (Life Technologies). Phase separation was achieved with the addition of chloroform (Sigma-Aldrich), and total RNA was precipitated from the aqueous layer with isopropanol (Sigma-Aldrich) using glycogen (Roche) as a carrier. RNA samples were sent to the Functional Genomics Center Zurich, where the RNA-Seq was performed. The TruSeq RNA Stranded sample kit (Illumina) was used to construct the sequencing libraries. In brief, total RNA samples (100 ng) were poly (A) enriched and reverse-transcribed into double-stranded cDNA, and TruSeq adapters were then ligated to double-stranded cDNA; then fragments containing TruSeq adapters on both ends were selectively enriched with PCR and subsequently sequenced on the Illumina Nova Seq. The fragments were mapped to the ensemble mouse reference genome GRCm38 (version 25.06.2015) using the STAR aligner (44). For normalization, the read counts were scaled with the use of the trimmed mean of M-values (TMM) method proposed by Robinson and Oshlack (45). PCA, matrix clustering, and the heatmap were generated using R. The mouse RNA-Seq data first reported in this study are available in NCBI’s Gene Expression Omnibus (GEO) under the accession numbers GSE140645 and GSE193537.

### Efferocytosis of apoptotic cells.

Thymocytes were isolated from mice and apoptosis was induced by exposure to 60 mJ/cm^2^ UV radiation (Spectrolinker XL-1500; Spectronics Corporation). After 2 hours of incubation at 37°C in IMDM plus 10% FCS, cells were labeled with 5 mM eFluor 670 (eBioscience) according to the manufacturer’s instructions and washed extensively with IMDM plus 10% FCS and PBS. Then, apoptotic cells (5 million cells in 50 μL PBS) were delivered i.t. to recipient mice. At 0.5, 2, and 22 hours after administration, efferocytosis by alveolar macrophages in the BAL and lung was assessed by flow cytometry.

### Influenza viral infection.

Influenza virus strain PR8 (A/Puerto Rico/34, H1N1) was originally provided by J. Pavlovic, University of Zurich. For infections, the mice were anesthetized and i.t. inoculated with indicated doses of virus in 50 μL endotoxin-free PBS. The temperature and weight of mice were monitored daily, and animals were euthanized if they fulfilled the severity criteria set out by institutional and cantonal guidelines.

### Measurement of O_2_ saturation.

The MouseOx pulse oximeter (Starr Life Sciences) was used to measure O_2_ saturation in influenza-infected mice on day 7 after infection. The depilatory agent was applied to the neck of mice 2–3 days prior to measurement to remove hair. Mice were sedated with 2.5 mg/kg i.p. midazolam (Roche) 0.5–1 hour before measurement. The sensor clip was placed on the neck and O_2_ saturation was measured each second over 3–5 minutes per mouse. Data shown is the average value of each mouse.

### Retroviral reconstitution of CSF2RA gene.

Two retroviral constructs based on Moloney murine leukemia virus, containing Csf2ra cDNA and GFP (RV*^Csf2ra-gfp^*) or GFP only (RV*^gfp^*), were used for transfection of retrovirus packaging cell line. Fresh viral supernatants containing nonreplicating retroviruses were used for transduction of fetal liver monocytes isolated as described above from *Csf2ra^–/–^* or CD45.1^+^ WT embryos. Cells were cultured in complete RPMI, supplemented with GM-CSF (30 ng/mL) for 7 days. All nontransduced fetal liver monocytes derived from *Csf2ra^–/–^* embryos could not proliferate. After a week, a homogenous population of receptor-expressing cells was detected and used for transfers. For WT cells, all live cells treated with the Csf2ra-overexpressing virus were used (irrespectively of the transduction level).

### Statistics.

Mean values and SD were calculated with GraphPad Prism. Student’s 2-tailed *t* test (unpaired) was used for comparing 2 groups, and ANOVA (1-way) was used for comparing multiple groups; *P* values of less than 0.05 were considered significant.

### Study approval.

All animal experiments were performed according to the guidelines of the Federal Animal Protection Act (TSchG) and the Federal Animal Protection Ordinance (TSchV) and were approved by the local animal ethics committee (Cantonal Veterinary Office, Zurich, license ZH054/18).

## Author contributions

FL, KMO, and MK designed the experiments. FL, KMO, and FP performed and analyzed experiments. LMP analyzed RNA-Seq data. CS and MK discussed data and provided conceptualization. FL, KMO, and MK wrote the manuscript.

## Supplementary Material

Supplemental data

## Figures and Tables

**Table 2:**
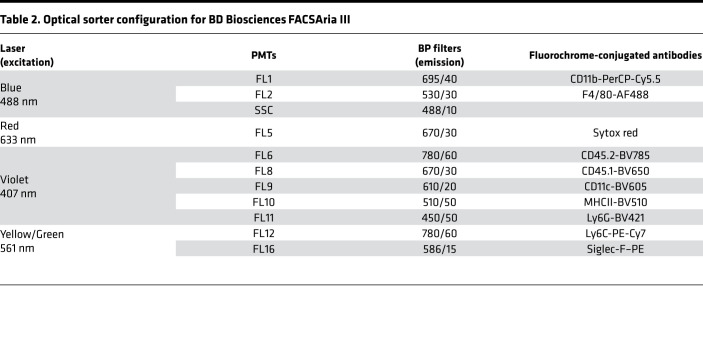
Optical sorter configuration for BD Biosciences FACSAria III

**Table 1:**
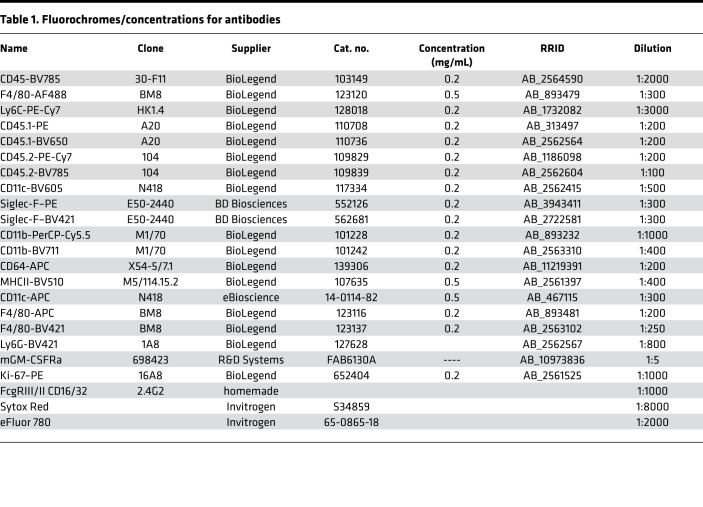
Fluorochromes/concentrations for antibodies

## Version 1 04/08/2022

Electronic publication
