# Supplementary material for: Gene therapy of Csf2ra deficiency in mouse fetal monocyte precursors restores alveolar macrophage development and function
Source: JCI Insight. 2022 Apr 8;7(7):e152271. doi: 10.1172/jci.insight.152271 (PMC9057586; doi:10.1172/jci.insight.152271)
Supplement: Supplemental data [file jciinsight-7-152271-s020.pdf]

Supplementary Materials

Gene therapy of *Csf2ra*-deficiency in mouse fetal monocyte precursors restores alveolar macrophage development and function

Fengqi Li[1, #], Katarzyna Maria Okreglicka[1, #], Federica Piattini[1], Lea Maria Pohlmeier[1], Christoph Schneider[1,2], Manfred Kopf[1, *]

[1]Institute of Molecular Health Sciences, Department of Biology, ETH Zürich, Switzerland.

[2]Institute of Physiology, University of Zürich, Switzerland.

[#]These authors contributed equally to this work.

*Correspondence author:

Address: Otto-Stern-Weg 7, 8093 Zürich, Switzerland;

Tel: +41 44 633 64 70;

E-mail: manfred.kopf@ethz.ch

This PDF file includes:

Figure S1-S8

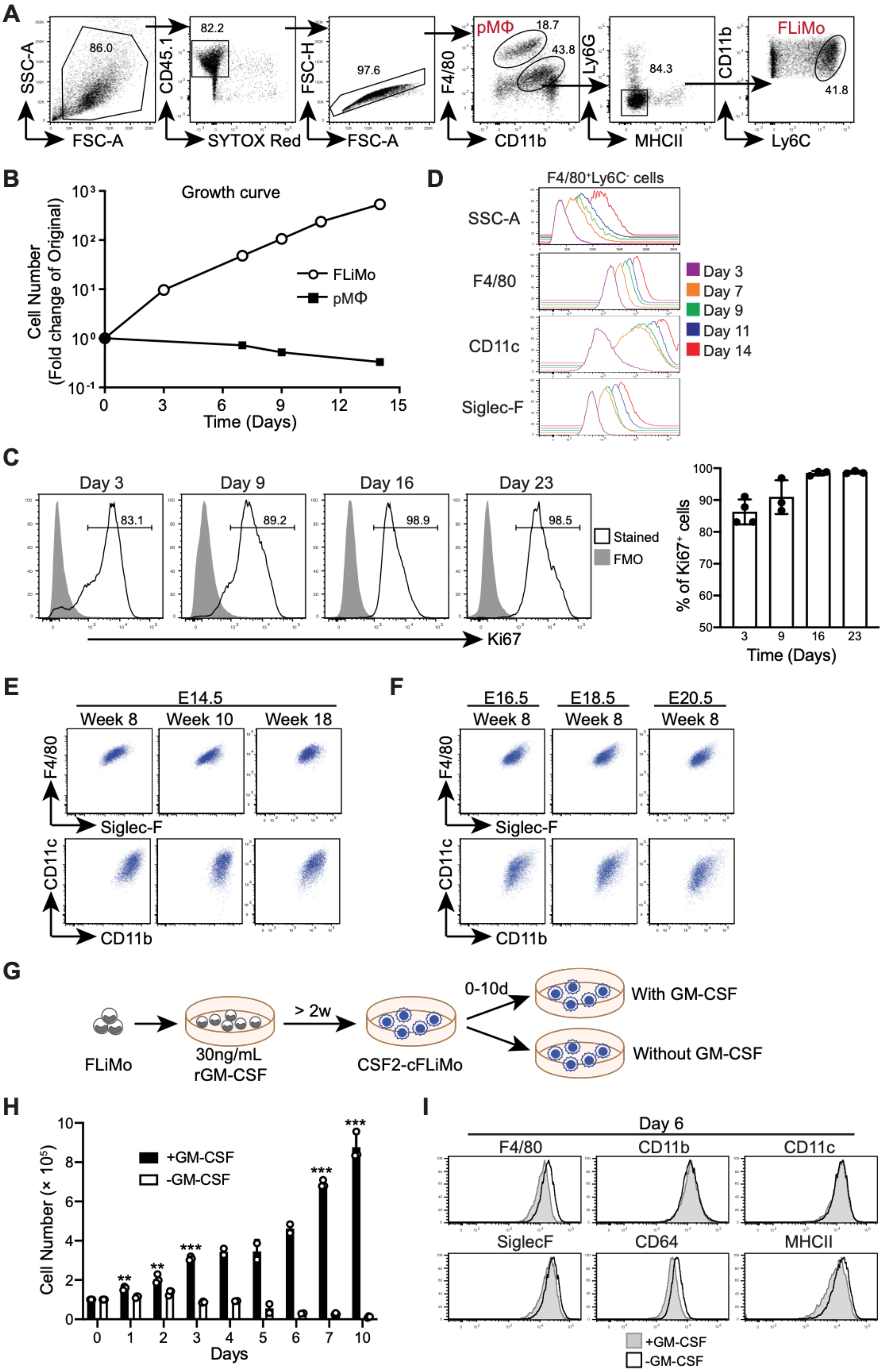
A
SSC-A
FSC-A
86.0
CD45.1
SYTOX Red
82.2
FSC-H
FSC-A
97.6
F4/80
CD11b
pMΦ
18.7
43.8
Ly6G
MHCII
84.3
CD11b
Ly6C
FLiMo
41.8
B
Growth curve
Cell Number (Fold change of Original)
Time (Days)
FLiMo
pMΦ
D
F4/80+Ly6C- cells
SSC-A
F4/80
CD11c
Siglec-F
Day 3
Day 7
Day 9
Day 11
Day 14
C
Day 3
Day 9
Day 16
Day 23
83.1
89.2
98.9
98.5
Stained
FMO
Ki67
% of Ki67+ cells
Time (Days)
E
E14.5
Week 8
Week 10
Week 18
F4/80
Siglec-F
CD11c
CD11b
F
E16.5
E18.5
E20.5
Week 8
G
FLiMo
30ng/mL rGM-CSF
> 2w
CSF2-cFLiMo
0-10d
With GM-CSF
Without GM-CSF
H
Cell Number (× 10^5)
+GM-CSF
-GM-CSF
Days
I
Day 6
F4/80
CD11b
CD11c
SiglecF
CD64
MHCII
+GM-CSF
-GM-CSF

Figure S1. FLiMo differentiate into long-lived, homogeneous, AM-like cells *in vitro* in the presence of GM-CSF (related to Figure 1)

(A) Sorting strategy for primitive macrophages (pMΦ) and FLiMo from fetal liver of 6-10 CD45.1 C57BL/6 embryos with mixed gender. Doublets and debris were excluded using FSC and SSC. FLiMo were identified as viable $CD45^{+}F4/80^{lo}CD11b^{int}MHCII^{-}Ly6G^{-}Ly6C^{+}$ cells and pMΦ were identified as viable $CD45^{+}F4/80^{hi}CD11b^{lo}$ cells. (B) Growth curves of FLiMo and pMΦ cultured *in vitro* with GM-CSF (30 ng/mL) (n=2/group). (C-F) Flow cytometry was used to characterize cultured cells. (C) Representative histograms of Ki67 expression in CSF2-cFLiMo at indicated timepoints showing FMO control (grey) and specific antibodies (black line), pre-gated on viable $CD45^{+}$ single cells (left panel). Percentages of $Ki67^{+}$ cells are shown in right penal (n=3 to 4/group). (D) Histograms for SSC-A, F4/80, CD11c and Siglec-F expression on $F4/80^{+}Ly6C^{-}$ cells at the indicated time points (n=2/group). (E-F) Representative dot plots of CSF2-cFLiMo from E14.5 embryos after 8, 10 and 18 weeks (E) or from E16.5, E18.5 and E20.5 embryos after 8 weeks *in vitro* (F), pre-gated on viable $CD45^{+}$ single cells (n=3/group). (G) Illustration of experimental regime. CSF2-cFLiMo cultured for more than 2 weeks in the presence of GM-CSF. The effects of GM-CSF deprivation were analyzed in H-I. (H) Numbers of CSF2-cFLiMo after removing GM-CSF (n=2 to 3/group). (I) Representative histogram of CSF2-cFLiMo 6 days after removing GM-CSF (n=2 to 3/group). Data are presented as mean ± SD and the results are representative of three experiments. Student's t test (unpaired) was used in H: ns, not significant; *$p < 0.05$, **$p < 0.01$, ***$p < 0.001$, ****$p < 0.0001$.

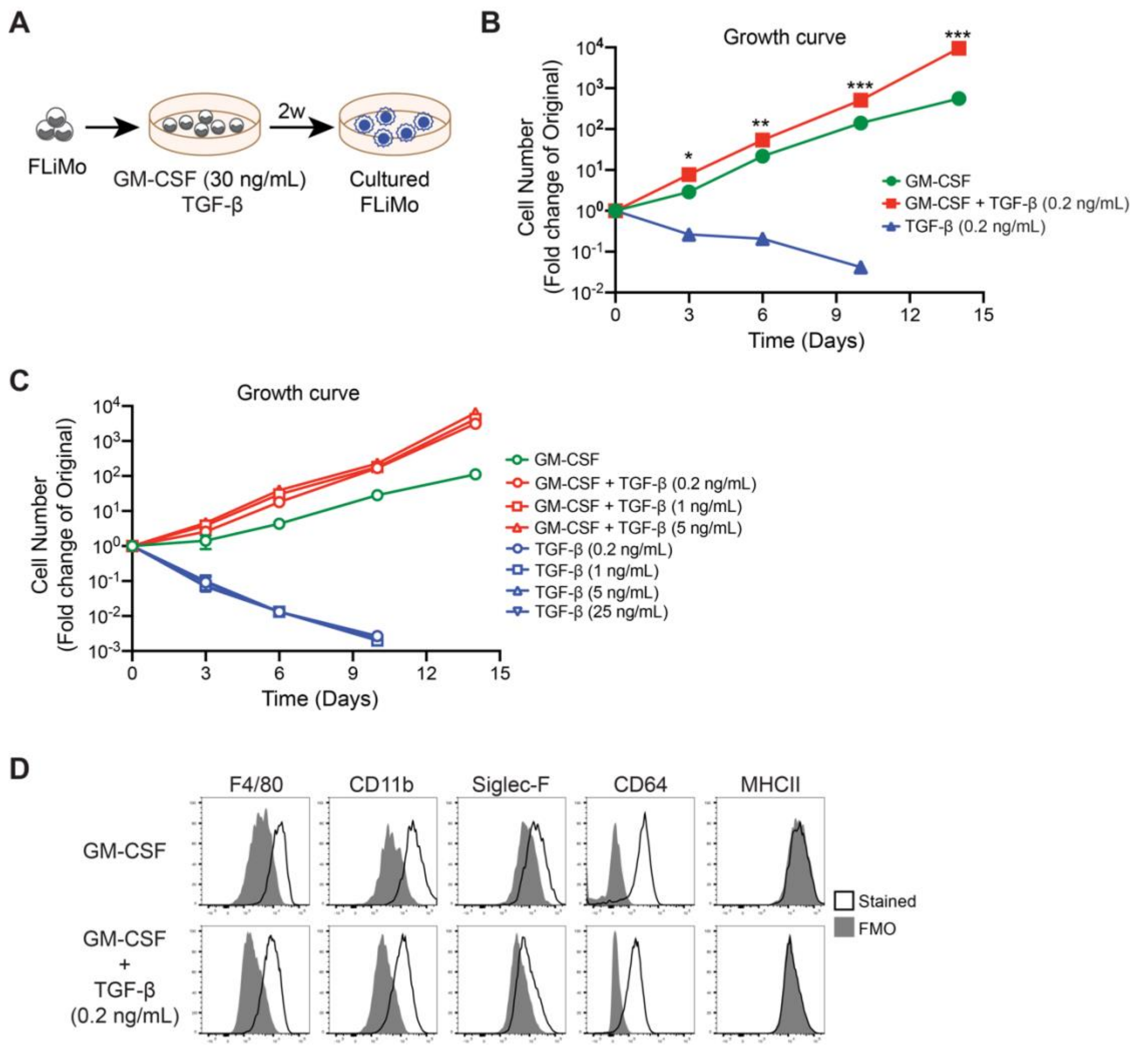

Figure S2. FLiMo culture with GM-CSF and TGF-β (related to Figure 1)

(A) Illustration of experimental regime. FLiMo were cultured *in vitro* with GM-CSF and/or TGF-β. (B) Growth curves of FLiMo cultured with 30ng/mL GM-CSF and/or 0.2ng/mL TGF-β (n=2/group). (C) Growth curves of FLiMo cultured with 30ng/mL GM-CSF and/or indicated concentration of TGF-β (n=2/group). (D) Phenotype of FLiMo 14 days after culturing with GM-CSF or GM-CSF + TGF-β (0.2ng/mL) (n=2/group). Data are presented as mean ± SD and the results are representative of two experiments. Student's t test (unpaired) was used in B to compare GM-CSF group and GM-CSF + TGF-β group: ns, not significant; *$p < 0.05$, **$p < 0.01$, ***$p < 0.001$, ****$p < 0.0001$.

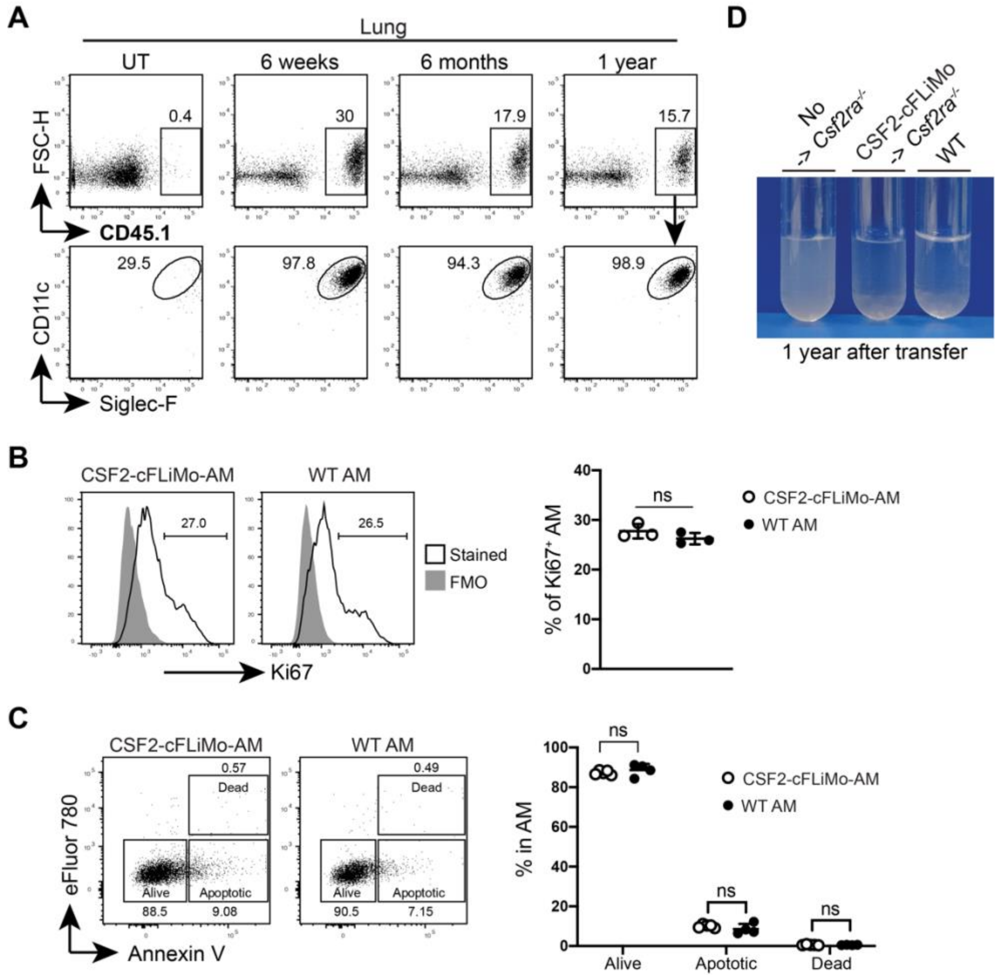

Figure S3. CSF2-cFLiMo have the potential to further differentiate into mature functional AM (related to Figure 1)

CSF2-cFLiMo generated from CD45.1 E14.5 embryos were transferred i.n. to CD45.2 *Csf2ra$^{-/-}$* mice and analyzed at the indicated time points as described in Fig. 1A. (A) Representative dot plots of donor-derived cells in the lung of recipients 6 weeks, 6 months and 1 year after transfer, pre-gated as viable single cells (n=3/group). (B) Representative histograms of Ki67 expression in CSF2-cFLiMo-derived AM in the BAL of recipients 3 weeks after transfer (left panel). Percentages of Ki67^{+} AM are shown in right penal (n=3/group). (C) Representative dot plots of Annexin V/eFluor 780 in CSF2-cFLiMo-derived AM in the BAL of recipients 3 weeks after transfer (left panel). Percentages of alive (double negative), apoptotic (Annexin V^{+}eFluor 780^{-}) and dead (double positive) AM are shown in right penal (n=5/group). 3-week- old WT mice were included as controls in B (n=3) and C (n=4). (D) Appearance of BAL fluid 1 year

after transfer (n=3/group). Age-matched untreated (UT) *Csf2ra*$^{-/-}$ and WT mice were included as negative and positive controls respectively in A and B (n=3/group). The data are representative of three experiments in A and D, two experiments in B and C. Data are presented as mean ± SD. Student's t test (unpaired) was used in B and C: ns, not significant; *$p < 0.05$, **$p < 0.01$, ***$p < 0.001$, ****$p < 0.0001$.

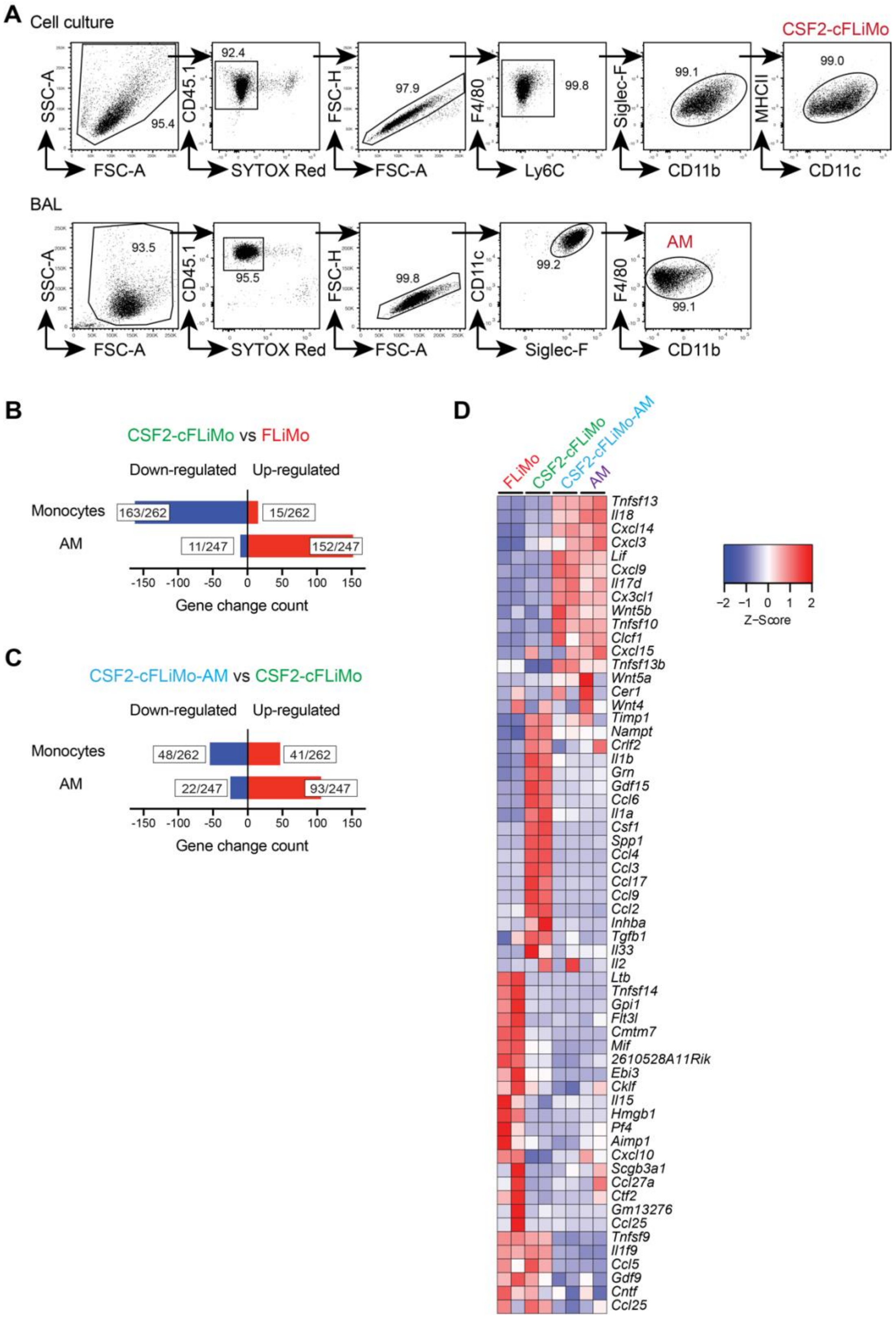
A
Cell culture
CSF2-cFLiMo
SSC-A
FSC-A
95.4
CD45.1
SYTOX Red
92.4
FSC-H
FSC-A
97.9
F4/80
Ly6C
99.8
Siglec-F
CD11b
99.1
MHCII
CD11c
99.0
BAL
SSC-A
FSC-A
93.5
CD45.1
SYTOX Red
95.5
FSC-H
FSC-A
99.8
CD11c
Siglec-F
99.2
F4/80
CD11b
AM
99.1
B
CSF2-cFLiMo vs FLiMo
Down-regulated
Up-regulated
Monocytes
AM
163/262
15/262
11/247
152/247
-150 -100 -50 0 50 100 150
Gene change count
C
CSF2-cFLiMo-AM vs CSF2-cFLiMo
Down-regulated
Up-regulated
Monocytes
AM
48/262
41/262
22/247
93/247
-150 -100 -50 0 50 100 150
Gene change count
D
FLiMo
CSF2-cFLiMo
CSF2-cFLiMo-AM
AM
Tnfsf13
Il18
Cxcl14
Cxcl3
Lif
Cxcl9
Il17d
Cx3cl1
Wnt5b
Tnfsf10
Clcf1
Cxcl15
Tnfsf13b
Wnt5a
Cer1
Wnt4
Timp1
Nampt
Crlf2
Il1b
Grn
Gdf15
Ccl6
Il1a
Csf1
Spp1
Ccl4
Ccl3
Ccl17
Ccl9
Ccl2
Inhba
Tgfb1
Il33
Il2
Ltb
Tnfsf14
Gpi1
Flt3l
Cmtm7
Mif
2610528A11Rik
Ebi3
Cklf
Il15
Hmgb1
Pf4
Aimp1
Cxcl10
Scgb3a1
Ccl27a
Ctf2
Gm13276
Ccl25
Tnfsf9
Il1f9
Ccl5
Gdf9
Cntf
Ccl25
−2 −1 0 1 2
Z−Score

Figure S4. Gene expression profiles of CSF2-cFLiMo *in vitro* and *in vivo* (related to Figure 3)

RNA sequencing was performed as described in Fig. 3A. (A) Sorting strategy for *in vitro* generated CSF2-cFLiMo, AM from CSF2-cFLiMo-reconstituted *Csf2ra*$^{-/-}$ mice or naive WT mice. Doublets and debris were excluded using FSC and SSC. CSF2-cFLiMo were identified as viable CD45^{+}F4/80^{+}CD11b^{+}Siglec-F^{+}CD11c^{+}MHCII^{-}Ly6C^{-} cells and AM were identified as viable CD45^{+}F4/80^{+}CD11b^{-}Siglec-F^{hi}CD11c^{hi} cells. (B-C) Bar graphs showing up-regulated and down-regulated (blue) genes from a comparison of CSF2-cFLiMo vs FLiMo (B) and CSF2-cFLiMo-derived AM vs CSF2-cFLiMo (C) (fold change > 2, P < 0.01, Fdr < 0.01) with gene signatures of monocytes and lung MΦ derived from Lavin *et al.* (2014) (n=2/group). (D) Heat maps showing expression of cytokine and chemokine genes in different.

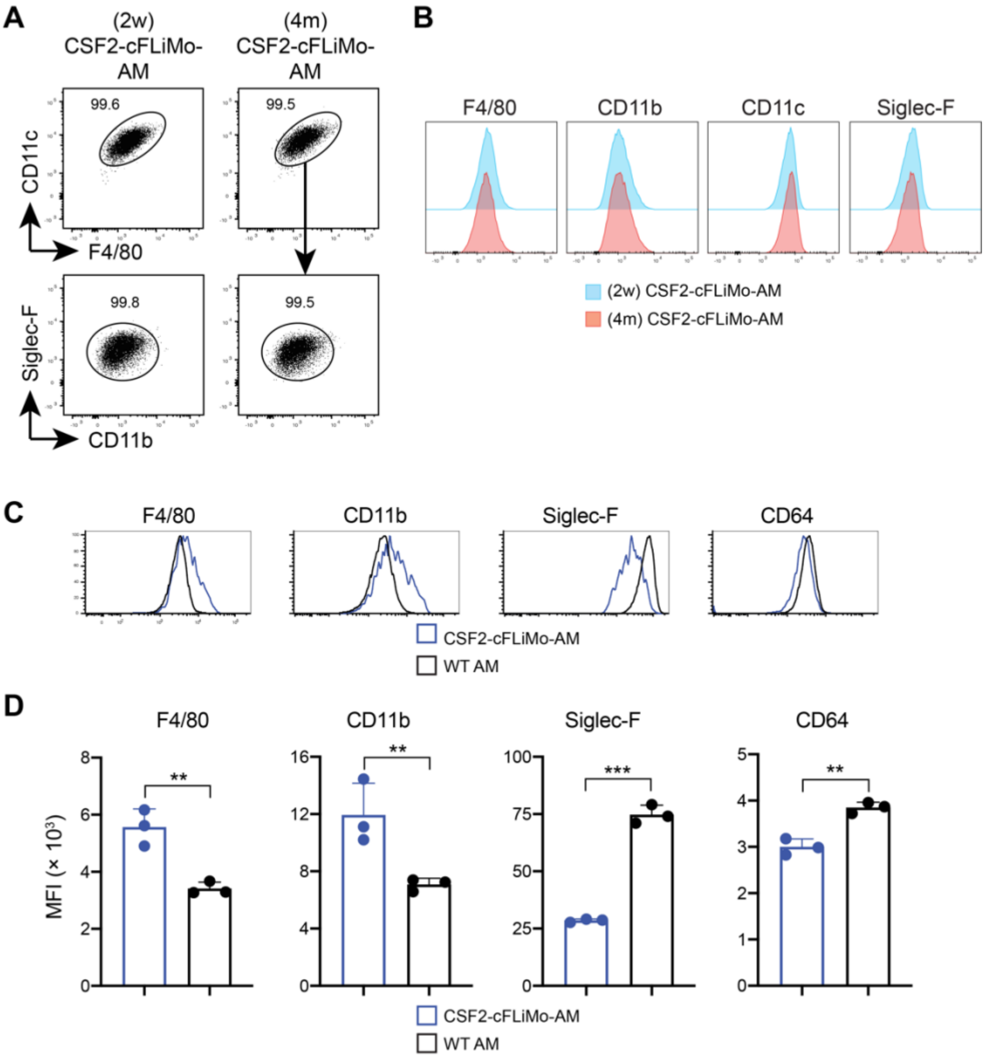

Figure S5. Transplantation of CSF2-cFLiMo to *Csf2ra*$^{-/-}$ mice (related to Figure 4)

(A-B) 5×10^4 of CD45.1 CSF2-cFLiMo generated from E14.5 embryos after 2-week or 4-month culture were transferred i.n. to neonatal CD45.2 *Csf2ra*$^{-/-}$ mice and analyzed 6 weeks later as described in Fig. 4A. Representative dot plots (A) and histograms (B) of donor-derived cells in the BAL of recipients, pre-gated as viable CD45.1$^+$ single cells (n=3/group). (C-D) CSF2-cFLiMo were transferred i.t. to adult *Csf2ra*$^{-/-}$ mice and analyzed 10 weeks later as described in Fig. 4E. (C) Representative histograms and (D) MFI of AM signature markers on CSF2-cFLiMo - derived AM and WT AM (n=3/group). The data are representative of three experiments. Data are presented as mean ± SD. Student's t test (unpaired) was used in D: ns, not significant; *p < 0.05, **p < 0.01, ***p < 0.001, ****p < 0.0001.

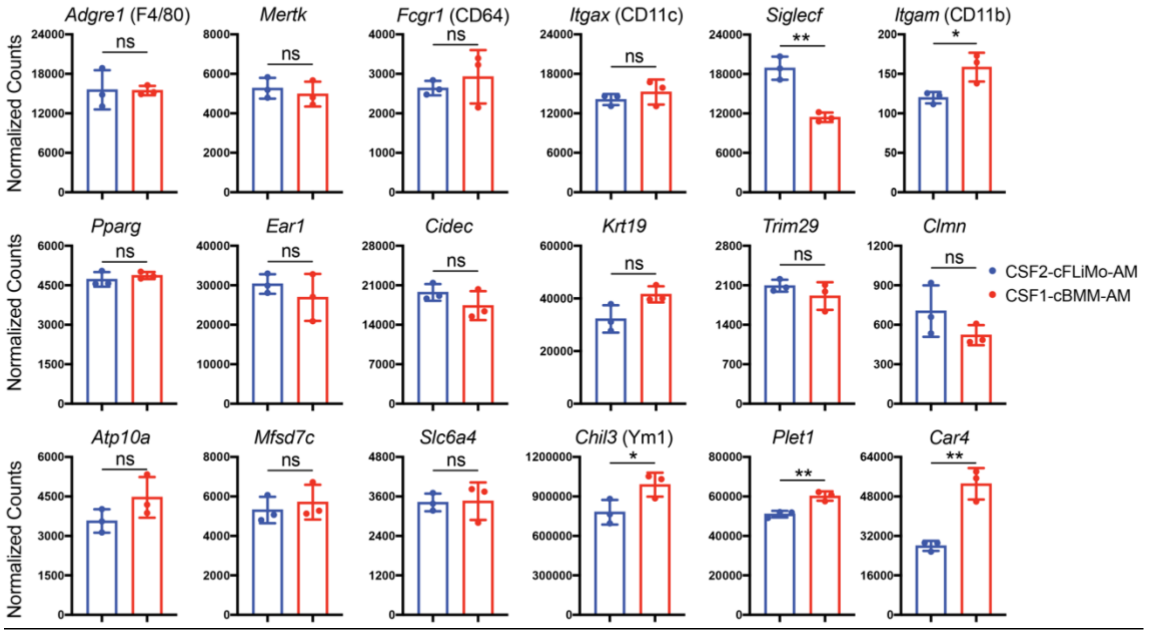

Figure S6. Minor differences in expression of AM markers and signature genes in CSF2-cFLiMo-derived AM and CSF1-cBMM-derived AM (related to Figure 5)

FLiMo from E14.5 embryos (CD45.1) and BM cells from adults (CD45.1) were cultured with GM-CSF (CSF2) for 2 weeks and M-CSF (CSF1) for 1 week, respectively, prior transfer to CD45.2 *Csf2ra*$^{-/-}$ neonates. CSF2-cFLiMo-derived AM and CSF1-cBMM-derived AM were sorted as viable CD45^{+}F4/80^{+}CD11b^{-}Siglec-F^{hi}CD11c^{hi} cells from the BAL 10 weeks later and gene expression determined by RNA sequencing (three biological replicates per group). Bar graphs showing expression indicated AM markers and signature genes. Data are presented as mean ± SD. Student's t test (unpaired) was used: ns, not significant; *$p < 0.05$, **$p < 0.01$, ***$p < 0.001$, ****$p < 0.0001$.

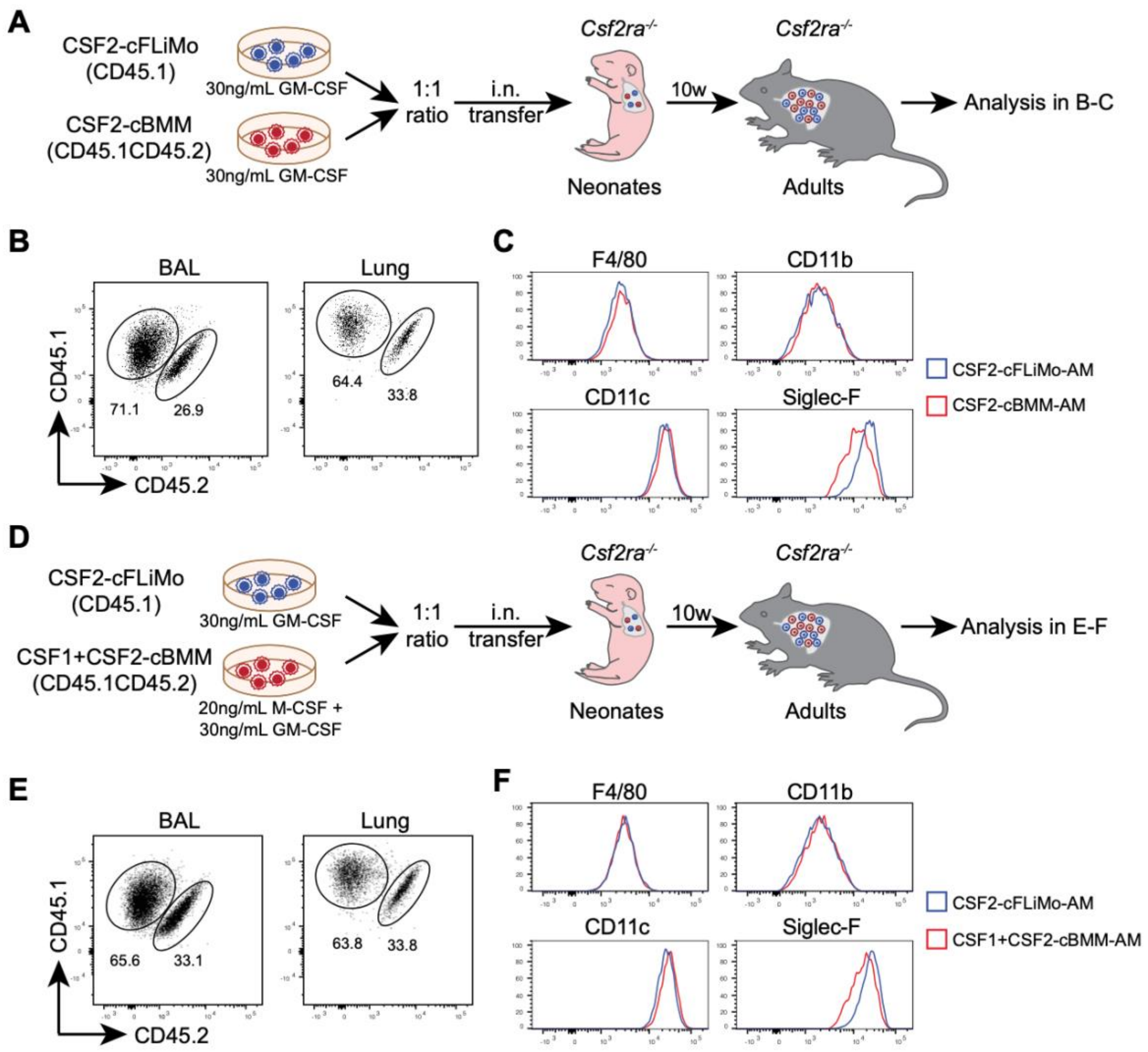

Figure S7. Competitive transplantation of BMM and CSF2-cFLiMo in *Csf2ra*$^{-/-}$ mice (related to Figure 5)

(A) Illustration of experimental regime. (B-C) FLiMo from E14.5 embryos (CD45.1) and bone marrow cells from adult mice (CD45.1CD45.2) were cultured for 2 weeks and 1 week, respectively, in the presence of GM-CSF. CSF2-cFLiMo and CSF2-cBMM were mixed in 1:1 ratio and transferred i.n. to *Csf2ra*$^{-/-}$ neonates (CD45.2) and analyzed 10 weeks later. (B) Representative dot plots of donor-derived AM in BAL and lung of the recipients (n=3/group). (C) Representative histograms showing expression of AM markers on CSF2-cFLiMo-derived AM and CSF2-cBMM-derived from BAL of recipients (n=3/group). (D-F) Illustration of experimental scheme with a design, performance, and analysis similar to Fig. S6A-C described

above with the exception that BM cells were grown in the presence of both M-CSF and GM-CSF *in vitro* prior mixture with CSF2-cFLiMo and transfer to *Csf2ra*$^{-/-}$ neonates (n=3/group).

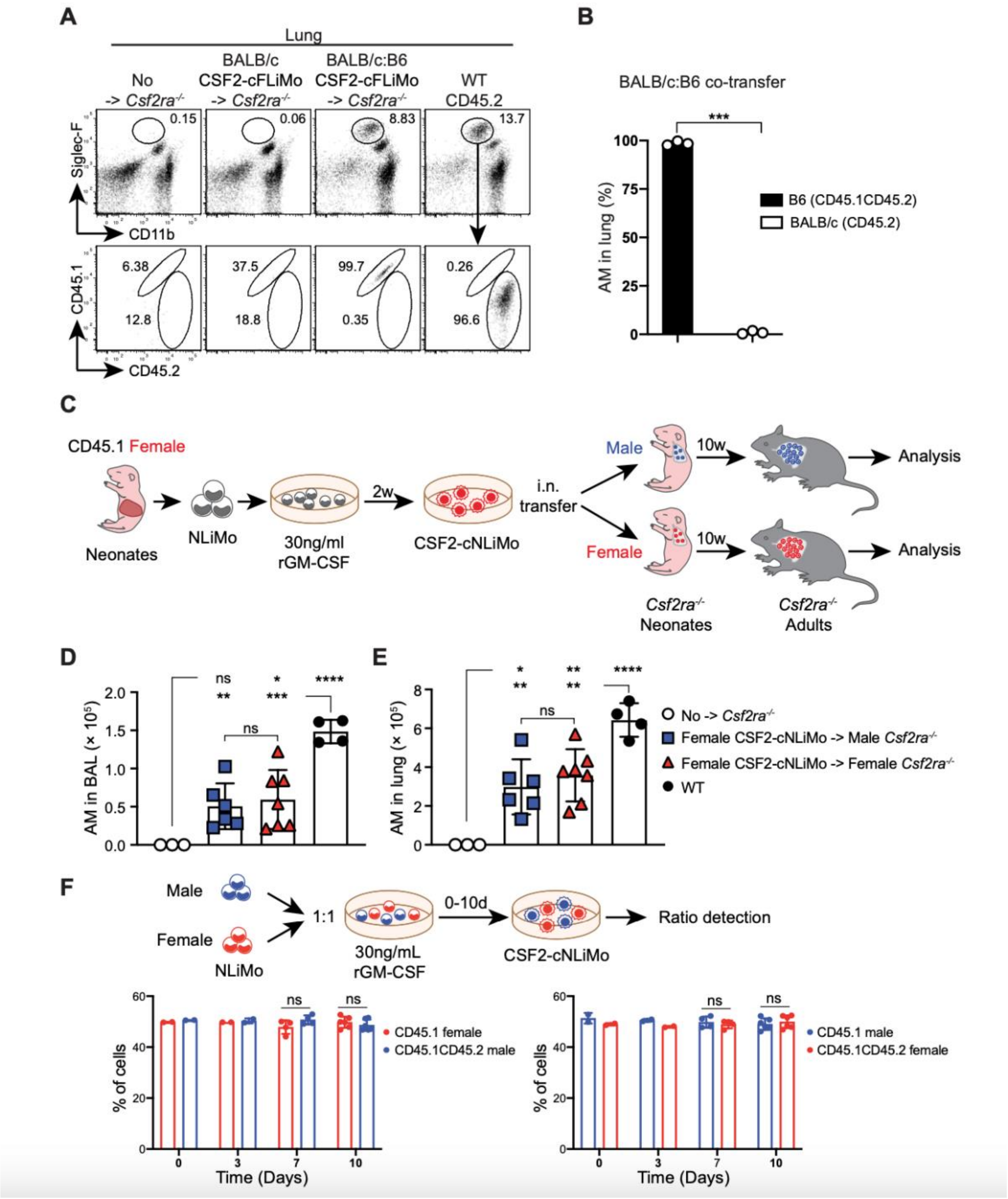

Figure S8. The MHC and gender compatibility in allogenic CSF2-cFLiMo transfers (related to Figure 6)

(A-B) BALB/c CSF2-cFLiMo were transferred alone or in a 1:1 mixture with C57BL/6 CSF2-cFLiMo to neonatal *Csf2ra*$^{-/-}$ mice and analyzed 10 weeks later as described in Fig. 6A. (A) Representative dot plots showing the phenotype of donor-derived AM in the lung, pre-gated

on viable CD45$^+$ single cells. Age-matched *Csf2ra*$^{-/-}$ and C57BL/6 (WT) mice were included as negative and positive controls (n=3/group). (B) Percentage of donor-derived AM in lungs of co-transferred recipients (n=3). (C) Illustration of experimental regime. CSF2-cNLiMo generated from CD45.1 WT female neonates were cultured for 2-week and transferred i.n. to neonatal CD45.2 *Csf2ra*$^{-/-}$ mice. Recipients were grouped according to gender and analyzed 10 weeks later in D-E. (D-E) Numbers of donor-derived AM (n=6 to 7/group) and WT AM in the BAL (D) and lung (E) are shown. Age-matched *Csf2ra*$^{-/-}$ (n=3) and CD45.2 WT (n=4) mice were included as negative and positive controls. (F) NLiMo were isolated from male and female neonates (CD45.1 or CD45.1/CD45.2), mixed and cultured in a 1:1 ratio in the presence of GM-CSF *in vitro*. Cell cultures were analyzed at indicated days. Bar graph shows the frequency of male and female cells (n=2 to 6/group). The data are representative of three experiments (A and B), and two experiments (C-F). Data are presented as mean ± SD. Student's t test (unpaired) was used in B, F and ANOVA (one way) was used in D, E: ns, not significant; *$p < 0.05$, **$p < 0.01$, ***$p < 0.001$, ****$p < 0.0001$.
